# Neutrophil myeloperoxidase as a functional biomarker for RSV severity: implications for in vitro therapeutic screening

**DOI:** 10.1038/s41467-026-74414-0

**Published:** 2026-07-13

**Authors:** Machaela Palor, Tereza Masonou, Elisabeth J. Robinson, Wenqing Chen, Samuel Ellis, Laura Buggiotti, Amy I. Jacobs, Thomas Benoist, Paolo De Coppi, Jennifer L. Rohn, Gabriele Pollara, Mario Cortina-Borja, Maximillian N. J. Woodall, Robert E. Hynds, Rosalind L. Smyth, Samiran Ray, Claire M. Smith

**Affiliations:** 1 Great Ormond Street Institute of Child Health, University College London, London, UK; 2 Department of Surgery and Cancer, Faculty of Medicine, Imperial College London, London, UK; 3 Clinical Research Department, London School of Hygiene and Tropical Medicine, London, UK; 4 Division of Medicine, University College London, London, UK; 5 Institute of Infection, Immunity & Transplantation, University College London, London, UK; 6 Epithelial Cell Biology in ENT Research (EpiCENTR) Group, Great Ormond Street Institute of Child Health, University College London, London, UK; 7 Paediatric Intensive Care Unit, Great Ormond Street Hospital for Children NHS Foundation Trust and NIHR Biomedical Research Centre, London, UK

**Keywords:** Infection, Neutrophils

## Abstract

Respiratory syncytial virus (RSV) is a leading cause of severe lower respiratory tract infections in infants, yet therapeutics are lacking. The aim of this study was to develop a pre‑clinical model that recapitulates key clinical outcomes in infants with RSV bronchiolitis, such as neutrophil activation and migration into the airways. Peripheral blood neutrophils from infants with severe RSV disease admitted to the Paediatric Intensive Care Unit showed elevated myeloperoxidase (MPO) in children with RSV, compared to age-matched controls. To mechanistically model this response, we established an air–liquid interface (ALI) system incorporating paediatric airway epithelial cells, endothelial cells and neutrophils from adults, to recapitulate the blood-airway barrier. Following RSV infection, with and without treatment with antivirals remdesivir or RSV604, neutrophil migration and activation were assessed using flow cytometry. While both drugs reduced viral load, only RSV604 attenuated MPO expression. This model suggests that MPO could be useful as a readout of therapeutic efficacy. Targeting neutrophil-driven inflammatory pathways may be critical for reducing pathology in infant RSV infection.

## Abstract (web summary)

Preclinical human models are needed to investigate mechanisms and evaluate therapies for severe respiratory syncytial virus (RSV) infection in children. The authors here develop a paediatric air–liquid interface (ALI) model incorporating airway epithelial and endothelial cells with neutrophils and show that the antiviral RSV604 reduces viral load and attenuates neutrophil myeloperoxidase activity.

## Introduction

Respiratory syncytial virus (RSV) is a leading cause of severe lower respiratory tract infections in infants and young children, resulting in approximately 3.2 million hospital admissions and an estimated 118,000 deaths in children under 5 years of age^1^. Despite these substantial health impacts, current therapeutic options primarily focus on prevention and symptom management. There are currently no routinely used, effective antivirals for immunocompetent children or adults, and ribavirin, historically the only licensed antiviral, is now rarely used even in immunocompromised populations due to high cost and concerns about toxicity^2,3^. Prophylactic strategies for high-risk infants include palivizumab, a monoclonal antibody requiring monthly dosing^4,5^ and nirsevimab, a recently approved monoclonal antibody shown to provide extended protection with a single dose^6,7^. The lack of accessible, effective therapies highlights the need for continued advancements in RSV treatment and prevention.

**Fig. 1:**
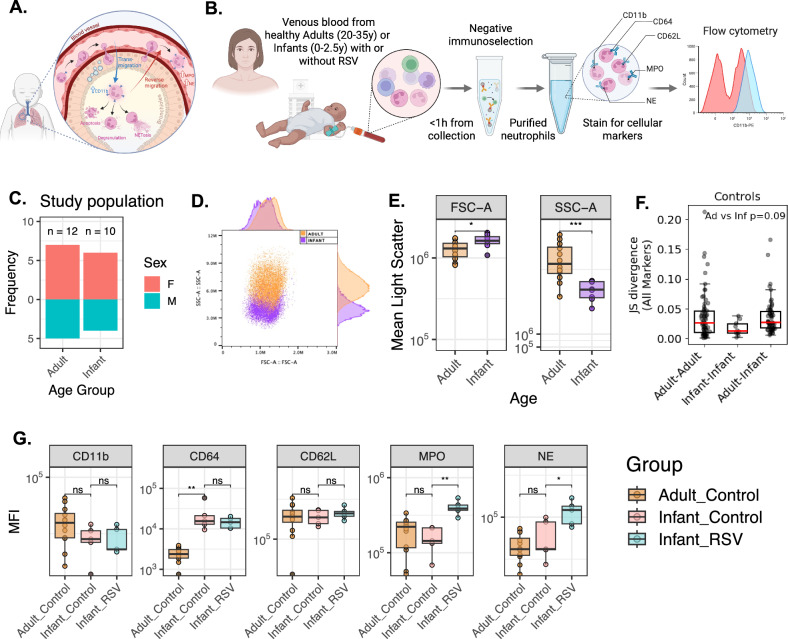
Phenotypic comparison of neutrophils from adults and infants with or without RSV infection. **A** Neutrophil trans-epithelial migration during RSV infection in the infant airway, highlighting neutrophil activation and degranulation. Created in BioRender. Woodall, M. (2026) https://BioRender.com/gt08btt**. B** Schematic of study design showing blood sampling from adults and infants with RSV bronchiolitis (RSV) and uninfected control ICU patients (DC). Created in BioRender. Woodall, M. (2026) https://BioRender.com/gt08btt**. C** Study population demographics by age group and sex (F female, M male). **D** Representative flow‑cytometry plots illustrating neutrophil gating and marker expression profiles. **E** Comparison of forward scatter (FSC‑A) and side scatter (SSC‑A) between adult and infant neutrophils, indicating age‑associated differences in cell size and granularity. Boxplots display median and interquartile range (*n* = 12 adult, *n* = 10 infants, biologically independent samples). * represents *p* < 0.05 ***p < 0.001 using unpaired t test. **F** Jensen–Shannon divergence (JSD) analysis of control donor by age. Boxplots display median and interquartile range. **G** Median fluorescence intensity (MFI) of neutrophil activation and granule markers (CD11b, CD64, CD62L, MPO and NE). Boxplots display median and interquartile range (*n* = 5 infant, *n* = 10 adult biologically independent samples). ns non-significance, *p < 0.05, **p < 0.01 using unpaired t test. Source data are provided in the Source data file.

Neutrophils are rapidly recruited to the lungs during RSV infection, playing a key role in the host defence mechanism by targeting the virus^8^. However, excessive neutrophil infiltration and activation can contribute to airway inflammation, epithelial damage, and disease severity^9,10^. While mouse studies have shown that neutrophil activation primarily occurs within the lung environment during RSV infection^11,12^, others have shown that neutrophils in the systemic circulation of RSV-infected children also exhibit high levels of activation markers, indicating that their activation is not limited to the lung environment^13,14^. Additionally, RSV proteins have been detected within these circulating neutrophils^15^, raising the possibility that some neutrophils, after interacting with virus-infected cells in the lungs, may re-enter the bloodstream (Fig. 1A). This process of reverse migration could have significant implications for systemic inflammation and viral dissemination.

Understanding neutrophil behaviour and activation during RSV infection, including during their migration across the airway epithelial barrier, is crucial for developing therapeutic strategies to mitigate pathological inflammation without compromising the antiviral response. Studies have shown that trans-epithelial migration is essential for neutrophil activation during RSV infection, leading to higher expression of activation markers and release of inflammatory mediators^11,12,16^. In vivo studies are limited by their complexity and ethical considerations, necessitating the use of in vitro models that closely replicate the human airway environment, which can differ between children and adults^17^. Differentiated airway epithelial cells (AECs) cultured at the air–liquid interface (ALI) provide a physiologically relevant platform to study neutrophil migration and the effect of antiviral treatments on this process. This approach is particularly valuable given the urgent need for predictive pre-clinical models that can accelerate the development and optimisation of antiviral therapies.

In this study, we aimed to develop an in vitro model that recapitulates key clinical outcomes of infants with RSV bronchiolitis. We analysed neutrophils from RSV-infected infants and validated our clinical observations in a human trans-epithelial ALI migration model^11,18^. To enhance physiological conditions, we incorporated a vascular endothelial cell (EC) layer alongside differentiated AECs, enabling us to study early infection dynamics and neutrophil phenotype in a more representative airway environment. Finally, we evaluated the effects of two repurposed antiviral therapies, remdesivir (RDV) (a broad-spectrum nucleoside analogue) and RSV604 (a small-molecule RSV fusion inhibitor), on neutrophil migration, priming and degranulation, to assess their potential in modifying immune responses during RSV infection.

Here, we establish a human trans-epithelial airway model incorporating both vascular endothelial and differentiated airway epithelial layers to examine neutrophil migration and activation during RSV infection and to link these responses to clinical phenotypes observed in infected infants. We demonstrate that neutrophils acquire a primed, degranulating phenotype during epithelial migration and that this response is significantly modulated by antiviral treatment, with reduced migration and inflammatory activation in the presence of remdesivir or RSV604. These findings identify neutrophil–epithelial interactions as a useful target for intervention and support the use of physiologically relevant human models to accelerate the development of therapies that limit immunopathology while preserving antiviral defence.

## Results

### Comparable peripheral blood neutrophil phenotypes across age, with higher neutrophil degranulation markers in RSV-infected infants

To examine age‑related differences in neutrophil phenotype and how these may be altered by RSV infection, we analysed peripheral blood neutrophils from healthy adults and infants, including both uninfected controls and RSV‑infected infants (Fig. 1B). Adults and infants were balanced by sex (Fig. 1C), with detailed donor characteristics provided in Table 1.

**Table 1:** Participant demographics

Age group	Patient ID	Ethnicity	Age range	Sex	Group	Relevant medical notes	Neutrophils (cells/ml blood
Child	1006	White British	>6 months– 3 years	F	RSV	Paediatric acute respiratory distress syndrome (PARDS on high support). Prior rhino/enterovirus adenovirus NPA currently negative. Started on low-dose steroids, favipiravir/ nitazoxanide/ribavirin after the sample was taken	8.50 × 10^5^
Child	1081	White British	2–3 months	F	RSV	Worsening type 2 respiratory failure, diagnosed as RSV bronchiolitis. Premature birth at 27 weeks	9.50 × 10^5^
Child	1082	White British	2–3 months	F	RSV	Bronchiolitis—RSV B positive, premature birth at 31 + 3 weeks PARDS large neck capillary haemangioma, large atrial septal defect	3.50 × 10^5^
Child	1083	Other mixed background	>6 months– 3 years	M	RSV	Bronchiolitis—RSV A/B positive, Ex 34 + 0/40 chromosomal abnormality, Pierre Robin sequence, cleft palate, previous grade 3 airway. Chronic liver disease, obstructive sleep apnea, nocturnal BiPAP (resumed 18/10, off oxygen since April 2023). Recent history of flu B Nissen fundoplication + gastrostomy non-obstructive RV rhabdomyoma (last echo October 2023, no compromise NEC post-natally—ileostomy and colostomy reversed (2021))	2.18 × 10^6^
Child	1084	Other white background	2–3 months	M	RSV	RSV A/B positive. Born at term, no neonatal concerns. Rhino/enterovirus positive	6.30 × 10^5^
Child	1085	White British	4–6 months	F	DC	Recurrent aspiration-related lung infections, currently none. VACTERL with oesophageal atresia, sample D2 post-repair. Chronic kidney disease, on long-term renal replacement	1.80 × 10^6^
Child	1086	White British	>6 months– 3 years	M	DC	Vein of galen malformation, D1 post 2nd embolization (not ventilated)	1.60 × 10^6^
Child	1087	Other mixed background	≤1 month	F	DC	Polycystic kidney disease, lung hypoplasia	3.00 × 10^6^
Child	1088	White British	≤1 month	F	DC	Atrioventricular septal defect, coarctation of the aorta (D1 post-coarctation repair and pulmonary artery band	2.80 × 10^6^
Child	1089	Asian British-Indian	2–3 months	F	DC	Lymphatic malformation. Post-sclerotherapy, not ventilated	2.40 × 10^6^
Adult	930	Asian	25–30 years	F	Healthy	n/a	nd
Adult	961	White British/Irish	20–25 years	F	Healthy	n/a	nd
Adult	964	White British/Irish	20–25 years	F	Healthy	n/a	nd
Adult	1000	White British/Irish	20–25 years	M	Healthy	n/a	nd
Adult	1001	Asian	25–30 years	F	Healthy	n/a	nd
Adult	1004	White British/Irish	20–25 years	F	Healthy	n/a	nd
Adult	1005	Asian	20–25 years	M	Healthy	n/a	nd
Adult	1008	White British/Irish	20–25 years	F	Healthy	n/a	nd
Adult	1077	White British/Irish	30–35 years	M	Healthy	n/a	nd
Adult	1078	White British/Irish	30–35 years	M	Healthy	n/a	nd
Adult	1079	Asian	25–30 years	M	Healthy	n/a	nd
Adult	1080	White British/Irish	25–30 years	F	Healthy	n/a	nd

*n/a* not applicable, *nd* not done.

Flow‑cytometric analysis revealed that infant neutrophils exhibited lower forward scatter (FSC) and side scatter (SSC) compared with adult neutrophils (Fig. 1D), indicating reduced cell size and granularity. These differences were consistent across donors (Fig. 1E) and align with previously described distinctions between neonatal/infant and adult granulocytes^12^. There was no difference in FSC or SSC between uninfected controls and RSV‑infected infants (Supplementary Fig. 4a)

To assess phenotypic similarity across age groups, we calculated the Jensen–Shannon divergence (JSD) using uninfected control donors only. JSD did not differ significantly between adults and infants (*p* = 0.09; Fig. 1F), indicating that the overall multivariate neutrophil phenotypes were broadly comparable at baseline. In controls, activation and granule marker expression was also largely similar across ages, with CD64 the only marker found to be significantly higher in infants at baseline (Fig. 1G). Relative to infant controls, age-adjusted analyses showed significantly higher MPO and NE expression (*p* < 0.05) in RSV‑infected infants, whereas CD11b, CD64 and CD62L remained unchanged (Fig. 1G and Supplementary Fig. 4b).

### RSV infection drives neutrophil phenotypic and transcriptional reprogramming in infants

**Fig. 2:**
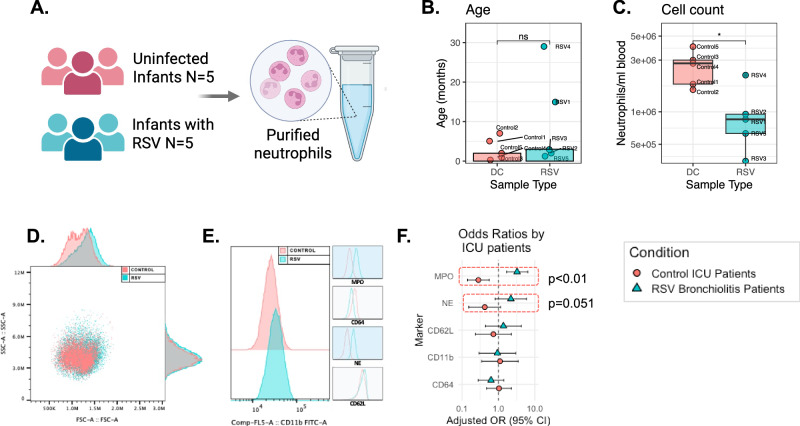
Neutrophil phenotyping in RSV‑infected infants. **A** Schematic of the method. Created in BioRender. Woodall, M. (2026) https://BioRender.com/gt08btt. **B** Age distribution of RSV‑infected infants and uninfected controls included in the study. ns represents non-significance using an unpaired Wilcoxon test. **C** Absolute neutrophil count in peripheral blood from control and RSV‑infected infants. Boxplots display median and interquartile range (*n* = 5 biologically independent samples per group) * represents *p* < 0.05 using an unpaired Wilcoxon test. **D**,** E** Flow‑cytometric analysis of circulating neutrophils. **D** Representative scatter plots illustrating the neutrophil gating strategy. **E** Representative histograms showing expression levels of key neutrophil surface markers. **F** Odds ratio analysis showing relative likelihood of marker expression changes in RSV versus control patients; for each marker, a generalised linear model with a quasibinomial error distribution were fitted to estimate the effect of condition on response probability, with exponentiated coefficients reported as odds ratios and 95% confidence intervals. (*n* = 5 biologically independent samples per group). Source data are provided in the Source data file.

We then profiled wider phenotypic changes in neutrophils from RSV‑infected infants and age‑matched uninfected controls (Fig. 2A, B). We found that peripheral blood neutrophil counts were significantly lower in RSV‑infected infants, with a mean viable cell count of 9.9 × 10⁵ cells/mL compared to 2.6 × 10⁶ cells/mL in control infants (*p* < 0.05) (Fig. 2C), consistent with enhanced tissue recruitment during infection^8,19,20^. Flow cytometric analysis (Fig. 2D) showed differences in neutrophil marker expression between the groups (Fig. 2E), and odds‑ratio analysis confirmed that MPO and NE were the markers most strongly enriched in RSV infection, indicating a significantly increased likelihood of elevated expression relative to controls (Fig. 2F).

**Fig. 3:**
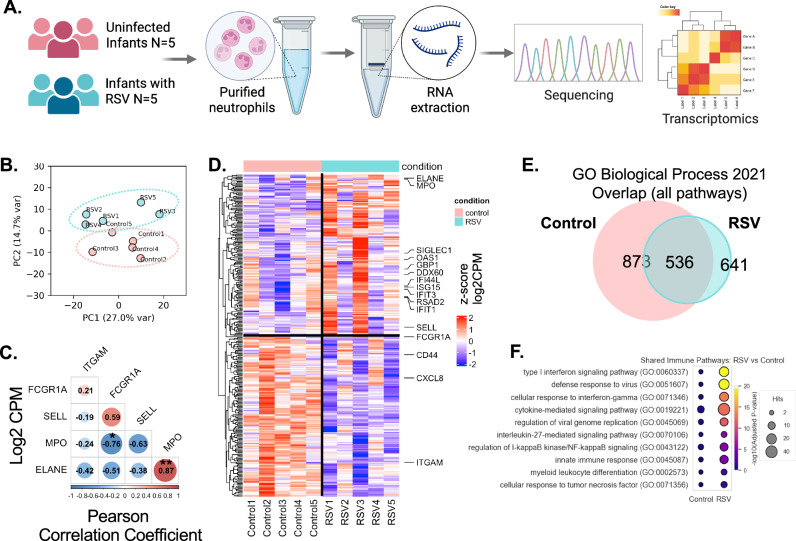
Transcriptomic profiling of neutrophils from RSV‑infected infants. **A** Schematic illustrating sample processing workflow, including neutrophil purification, RNA extraction and transcriptomic analysis. Created in BioRender. Woodall, M. (2026) https://BioRender.com/gt08btt. **B** Principal component analysis (PCA) of neutrophil transcriptomes from RSV‑infected (*n* = 5) and control infants (*n* = 5), demonstrating clear separation by PC2 based on infection status. **C** Correlation matrix of transcript levels of key neutrophil marker genes. Data derived from Pearson correlation coefficients between selected log transformed normalised gene counts; *p < 0.05, **p < 0.01. **D** Heatmap of differentially expressed genes (DEGs) between RSV and control neutrophils. Rows represent individual genes, columns represent donors and colours indicate z‑score-scaled expression. Highlighted genes denote strongly up- or down‑regulated transcripts. **E** Venn diagram of gene ontology (GO) enrichment analysis of DEGs, showing number of shared pathways associated with control or RSV neutrophils. **F** Dot‑plot illustrating the top enriched GO biological process pathways derived from differentially expressed genes. Dot size represents the number of genes contributing to each pathway, and dot colour indicates adjusted *p*‑value. Pathways are grouped according to enrichment in RSV‑infected or control neutrophils. Source data are provided in the Source data file.

To understand infection‑driven changes beyond cell protein expression, we performed transcriptomic profiling of purified neutrophils (Fig. 3A). Principal component analysis (PCA) revealed a clear separation between RSV‑infected and control infants (Fig. 3B), indicating an infection‑associated transcriptional programme. Gene expression correlation analysis across both conditions showed significant correlation between *ELANE* (encoding NE) and *MPO* (encoding MPO) (Fig. 3C). Differential expression analysis identified RSV‑responsive genes (Fig. 3D), and gene ontology enrichment revealed 105 pathways uniquely associated with RSV infection and 536 pathways shared between groups (Fig. 3E). RSV induced marked up‑regulation of antiviral, interferon‑stimulated, cytokine‑responsive and inflammatory pathways compared to control neutrophils (Fig. 3F). Together, these findings highlight a broad transcriptional reprogramming of circulating neutrophils in response to RSV infection.

### RSV infection increases neutrophil migration and activation in a paediatric airway epithelium model

**Fig. 4:**
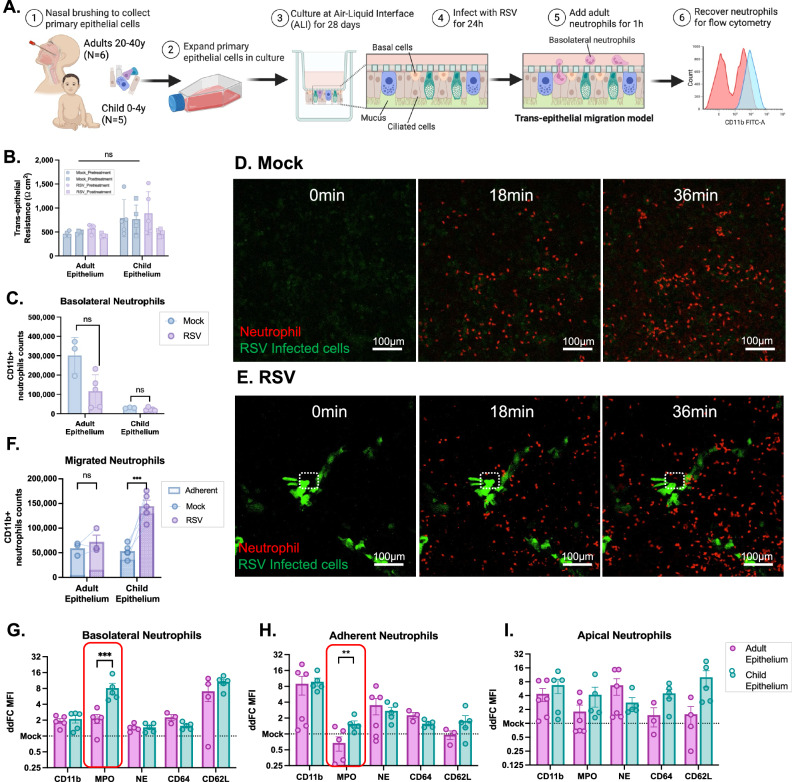
RSV infection enhances neutrophil migration across paediatric airway epithelium and alters neutrophil marker expression. **A** Schematic of experimental workflow. Created in BioRender. Woodall, M. (2026) https://BioRender.com/gt08btt. **B** Trans‑epithelial electrical resistance (TEER) of adult and child ALI cultures under mock or RSV‑infected conditions, measured prior to neutrophil assays, before and after mock or RSV treatment. Bars show mean ± SEM. ns not significant. (*n* = 5 child and 4 adult biological samples per group). **C** Quantification of basolateral neutrophils in adult or child ALI cultures under mock or RSV infection. Bars show mean ± SEM; (*n* = 5 child and 3 adult biological samples per group). Statistical analysis was performed using two‑way ANOVA with Bonferroni correction. ns non-significance. **D&E** Image still from timelapse videos (see Supplementary Videos 1 and 2) showing neutrophils (red) interacting with **D** mock‑infected and **E** RSV‑infected epithelial cultures. RSV‑infected epithelial cells are shown in green. Imaged every 2 min for 1 h from the addition of neutrophils using an inverted Zeiss LSM 710 confocal microscope. Scale bars, as indicated. White boxed area indicates zone showing of loss of GFP-positive cell. **F** Quantification of migrated neutrophils following migration across adult or child ALI cultures under mock or RSV infection. Bars show mean ± SEM; statistical analysis was performed between mock and RSV conditions using a paired two‑way ANOVA with Bonferroni correction. ns non-significance, ***p < 0.001. (*n* = 5 child and 3 or 6 adult biological samples per group). **G**–**I** Flow‑cytometric characterisation of neutrophil activation following migration across adult or child epithelium. Expression of CD11b, MPO, NE, CD64 and CD62L is shown as ΔMFI relative to mock infected controls for neutrophils collected from **G** basolateral, **H** adherent and **I** apical compartments. Bars show mean ± SEM (*n* = 5 child and 6 adult biological samples per group); statistical analysis was performed between mock and RSV conditions using a paired two‑way ANOVA with Bonferroni correction, **p < 0.01, ***p < 0.001. Only those with *p* < 0.05 are shown in (**G**–**I**). Source data are provided in the Source data file.

To test whether age-associated interactions with the airway microenvironment drives the heightened neutrophil degranulation observed in blood, we infected well‑differentiated air–liquid interface (ALI) nasal epithelial cultures from children and adult donors with RSV or mock control (Fig. 4A). After 24 h infection, transepithelial electrical resistance (TEER) was not reduced by RSV infection relative to mock, with no loss of barrier integrity in either age group (Fig. 4B).

We then applied a trans‑epithelial migration assay in which purified adult neutrophils were added to basolateral side for 1 h. Timelapse fluorescence imaging demonstrated increased accumulation of neutrophils at apical sites of RSV infection (Fig. 4D, E). Quantitatively, RSV infection increased neutrophil transmigration, with a greater number migrating across the child epithelium than adult epithelium (Fig. 4F). This suggests that the paediatric airway induces stronger recruitment signals during RSV infection. Notably, basolateral neutrophil numbers did not differ between age groups (Fig. 4C).

We next profiled neutrophil phenotypes after exposure to RSV‑infected paediatric and adult epithelium by flow cytometry. Here, basolateral neutrophils exposed to RSV-infected paediatric epithelium displayed higher expression of the degranulation marker MPO compared to the respective adult. Other markers, such as NE, CD11b, CD64 and CD62L, remained largely unchanged between age groups (Fig. 4G). A similar pattern was observed in adherent neutrophils at the epithelial interface (Fig. 4H), with paediatric epithelium consistently displaying higher MPO expression relative to mock and compared to the respective adult epithelium. No differences in neutrophil marker expression were observed in apical neutrophils recovered after full transmigration of the epithelial layer (Fig. 4I). Together, these results indicate that RSV‑infected paediatric epithelium drives both enhanced neutrophil recruitment and selective degranulation, aligning with the higher MPO/NE expression observed in infant blood.

### Endothelial cells modulate cytokine response and neutrophil migration without affecting RSV replication

**Fig. 5:**
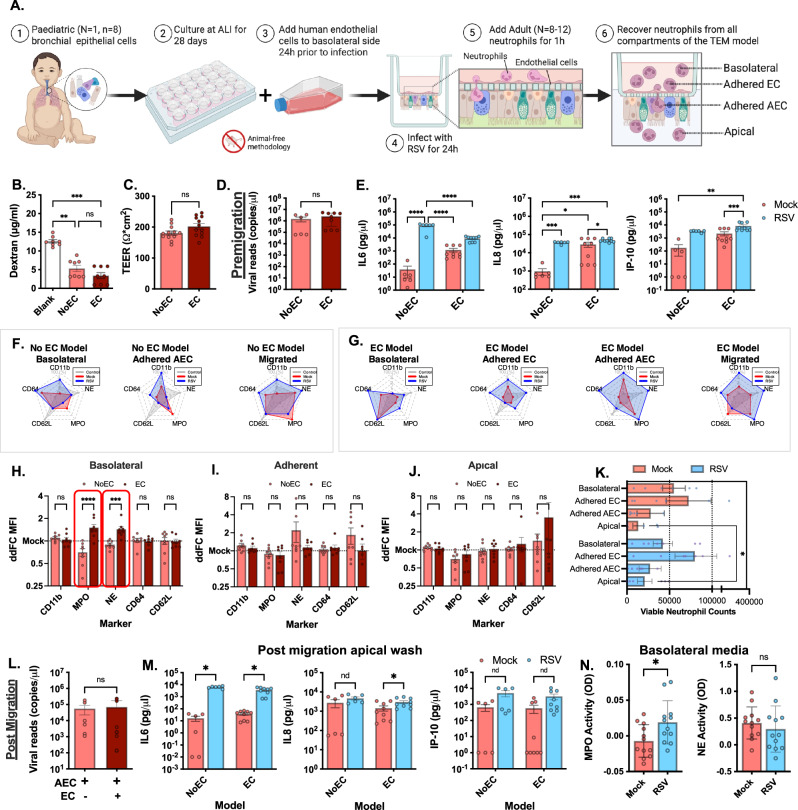
Endothelial–epithelial crosstalk shapes neutrophil responses to RSV infection in a co-culture airway model. **A** Schematic of the different co-culture models. Created in BioRender. Woodall, M. (2026) https://BioRender.com/gt08btt. **B–E** Barrier function and cytokine responses of AEC monocultures (NoEC) and AEC+EC cocultures (EC) under mock or RSV conditions. Statistical analysis was performed between mock and RSV conditions using a paired Kruskal–Wallis with Dunn's correction. **B** FITC‑dextran permeability. Bars show mean ± SEM (*n* = 8 independent experiments). Statistical analysis was performed between all conditions using a paired Kruskal–Wallis with Dunn's correction. **C** TEER. Bars show mean ± SEM (*n* = 12 independent experiments). Statistical analysis was performed between NoEC and EC conditions using a unpaired T test; **D** viral reads mean ± SEM (*n* = 6–9 independent experiments). Statistical analysis was performed between NoEC and EC conditions using a unpaired T test; **E** IL‑6, IL‑8 and IP-10 secretion from NoEC or EC cultures under mock or RSV infection. Bars show mean ± SEM. (*n* = 6 NoEC, *n* = 9 EC independent experiments). Statistical analysis was performed between mock and RSV conditions and NoEC and EC conditions using a mixed-effects model and Bonferroni test for multiple comparisons. **F**,** G** Radar plots showing quartile scores for neutrophil activation markers after migration through mock‑ or RSV‑infected cultures, as determined by flow cytometry. The centre of each plot represents the lowest level of marker expression, while points closer to the edges indicate higher expression levels. Flow cytometric analyses of neutrophils recovered from NoEC vs EC models under RSV infection (data shown is ΔΔFC of MFI relative to medium-only and mock controls) across the **H** basolateral, **I** adherent and **J** apical compartments. Bars show mean ± SEM (*n* = 8 biologically independent neutrophil samples). Statistical analysis was performed between NoEC and EC conditions using an unpaired two‑way ANOVA with Bonferroni correction. ns non-significance, ***p < 0.001, ****p < 0.0001. **K** Total viable neutrophil counts recovered from each compartment. Bars show mean ± SEM (*n* = 8 biologically independent neutrophil samples); statistical analysis was performed between mock and RSV condition using Wilcoxon signed rank test, *p < 0.05. **L** Viral reads post-migration, Bars show mean ± SEM (*n* = 6–9 independent experiments). **M** Neutrophil‑derived factors in post‑migration basolateral supernatant from mock‑ or RSV‑exposed co-cultures: MPO (ng/mL), IL‑8 (pg/mL), IL‑1β (pg/mL). ns not significant, *p < 0.05, **p < 0.01, ***p < 0.001* and ****p < 0.0001. Bars show mean ± SEM (*n* = 8 biologically independent neutrophil samples). Statistical analysis was performed between mock and RSV conditions and NoEC and EC conditions using a mixed-effects model and Bonferroni test for multiple comparisons. Only significant effects are shown, *p < 0.05. **N** MPO and NE enzyme activity in basolateral supernatants. Statistical analysis was performed between mock and RSV conditions using a paired T test. Only significant effects are shown, *p < 0.05. Source data are provided as a Source data file.

Next, we aimed to establish a more physiologically relevant airway microenvironment by incorporating a vascular endothelial layer to simulate neutrophil extravasation during RSV infection (Fig. 5A). Initial experiments demonstrated that introducing ECs into AEC cultures significantly decreased dextran permeability across the membrane insert compared to empty wells (Fig. 5B) and preserved TEER values around 200 Ω cm^2^ (Fig. 5C). These findings suggest that the addition of an EC layer restricts passive permeability between the basolateral and apical compartments. Viral load measurements revealed no significant difference between conditions with or without ECs, indicating that ECs do not influence RSV replication (Fig. 5D). However, the cytokine response, including key mediators of inflammation and neutrophil migration, IL-6, IL-8 and IP-10, was significantly higher in apical supernatants from EC+AEC co-cultures at baseline (mock) compared to cultures lacking ECs. RSV infection further increased the cytokine response from baseline, irrespective of EC co-culture conditions (Fig. 5E).

### Endothelial cells heighten degranulation marker expression and the cytokine response following trans-epithelial migration during RSV infection

Analysis of neutrophil markers by flow cytometry indicated different expression patterns of neutrophil markers across different compartments of the model and infection conditions (Fig. 5F, G). Across both models (no EC and EC), RSV infection (blue) consistently induces the highest quartile scores for neutrophil marker expression compared to baseline (grey) or mock conditions (red). Paired analysis of neutrophil markers revealed that MPO and NE expression was significantly increased in neutrophils recovered from the basolateral compartment in the presence of ECs (Fig. 5H–J and Supplementary Fig. 6a–c). This corresponded with enhanced neutrophil migration, resulting in a greater number of viable neutrophils detaching from the EC+AEC model and migrating to the apical compartment (Fig. 5K and Supplementary Fig. 6d).

Viral load measurements post-migration showed no significant differences between non-EC and EC co-culture conditions, indicating that neutrophil transendothelial migration does not influence baseline viral replication dynamics (Fig. 5L and Supplementary Fig. 6e, f). In contrast, cytokine analysis demonstrated significantly higher concentrations of IL-6 and IL-8 following RSV infection (Fig. 5M). Supporting these findings, we also detected significantly higher MPO activity in the basolateral supernatant during RSV infection compared to mock controls (Fig. 5N). Together, these data suggest that endothelial‑mediated priming of neutrophils enhances their degranulation potential and contributes to an amplified inflammatory response following trans‑epithelial migration during RSV infection.

### Higher basolateral MPO and NE in response to RSV infection is dependent on direct contact with RSV-infected AECs

**Fig. 6:**
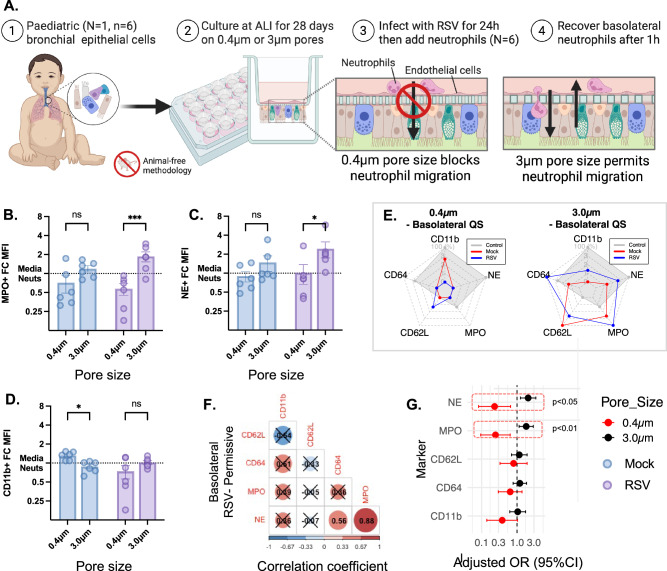
Neutrophil marker expression in response to RSV Infection in the basolateral compartment when neutrophils are permitted to migrate, or migration is blocked. **A** Schematic of methods created in BioRender. Woodall, M. (2026) https://BioRender.com/gt08btt. **B**–**D** Relative changes in neutrophil activation marker expression, shows as ΔΔ Fold change FC in median fluorescence intensity (MFI) for individual markers (MPO, NE. CD11b)) on neutrophils recovered from basolateral compartment under permissive (3.0 μm) and blocked (0.4 μm) pore size conditions. Bars show mean ± SEM from biologically independent neutrophil samples (*n* = 6 per condition). **E** Radar plots showing quartile scores (QS) of neutrophil activation markers (CD11b, CD62L, CD64, MPO and NE) under permissive (3.0 μm) and blocked (0.4 μm) conditions (*p < 0.05 compared to mock), illustrating coordinating changes in activation profiles. **F** Correlation matrix of marker expression under permissive RSV conditions. Positive (red) and negative (blue) correlations are displayed; with correlation strength indicated by circle size and colour intensity; non-significant correlations are marked with an X. **G** Odds ratio plot showing the relative likelihood of marker expression changes under RSV compared with mock conditions in the basolateral compartment for each pore size, either 0.4 or 3.0 μm pore sizes. Points indicate odds ratios with 95% confidence intervals. Red boxes indicate statistically significant changes (NE, *p* < 0.05; MPO, *p* < 0.01) between pore sizes. Statistical significance was assessed using a paired t-test (3.0 μm vs 0.4 μm pore size), unless otherwise indicated; ns not significant; *p < 0.05, **p < 0.01. Source data are provided in the Source data file.

To investigate whether the higher expression of MPO on basolateral neutrophils was driven by soluble factors from ECs or RSV-infected AECs, and was independent of neutrophil trans-epithelial migration, AECs were cultured on membranes with either permissive (3.0 µm pores) or blocked (0.4 µm pores) inserts (Fig. 6A). The smaller pores prevent neutrophil migration while still permitting passive diffusion of soluble factors.

Using this system, basolateral neutrophils exhibited significantly higher MPO (Fig. 6B and Supplementary Fig. 7a) and NE expression (Fig. 6C) in RSV-infected cultures under permissive (3.0 µm) conditions compared to blocked (0.4 µm) conditions (*P* < 0.05). No change in CD11b expression was detected with RSV infection (Fig. 6D). Radar plot analysis further showed that neutrophils in the blocked (0.4 µm) model exhibited lower activation scores across all markers compared to permissive cultures (Fig. 6E).

A correlation analysis demonstrated significant positive associations between MPO and NE in basolateral neutrophils following migration across RSV-infected cultures under permissive (3.0 µm) conditions (Fig. 6F and Supplementary Fig. 7b, c), suggesting co-expression of these markers. These findings suggest that increased MPO and NE in basolateral neutrophils is dependent on direct contact with RSV-infected AECs, rather than solely due to soluble infection-related factors. The odds ratio analysis (Fig. 6G) supports this interpretation, revealing that MPO and NE expression is dependent on neutrophil migration and shows a marked separation of odds ratios between pore sizes.

### Antiviral treatments reduce viral load but not inflammatory cytokine production

**Fig. 7:**
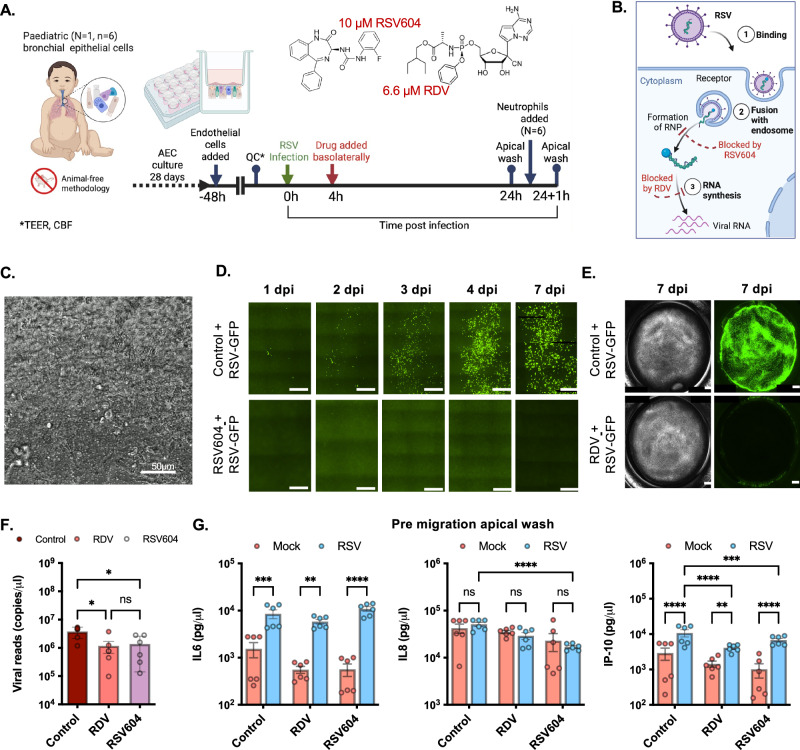
Effects of antiviral treatments on viral load and inflammation using in vitro RSV models. **A** Schematic of experimental design showing epithelial infection with RSV, followed by basolateral treatment with antivirals (RSV604 or Remdesivir [RDV]) and subsequent neutrophil migration. Created in BioRender. Woodall, M. (2026) https://BioRender.com/gt08btt. Chemical structures of RSV604 and RDV also shown, including the final concentrations for each antiviral, as previously reported. **B** Proposed mechanisms of action of RSV604 and RDV during the RSV life cycle. **C** Still image from high-speed video (Supplementary Video 3) demonstrating ciliary motility and epithelial function. **D** Representative fluorescence micrographs showing inhibition of RSV-GFP infection in airway epithelial cells (AECs) up to 7 days post-infection (dpi) following treatment with 10 μM RSV604. Scale bar = 300 μm. Images are representative from at least 3 independent experiments**. E** Representative whole-well scans (brightfield, grayscale; GFP, green) demonstrating suppression of RSV-GFP infection at 7 dpi with 6.6 μM RDV. Scale bar = 10 mm. Images are representative from at least 3 independent experiments. **F** Pre-migration viral load in apical supernatants (copies/μl). RSV604 and RDV significantly reduced viral load compared to RSV alone (*p* < 0.05). Bars represent mean ± SEM (*n* = 6 biologically independent neutrophil samples). **G** Pre-migration apical cytokine levels (log2 fold change from mock) for IL-6, IL-8 and IP-10. RSV infection significantly increased cytokine production; with further modulation observed following RSV604 and RDV treatment (*p* < 0.05). Bars show mean ± SEM (*n* = 6 biologically independent neutrophil samples); ns not significant; *p < 0.05, **p < 0.01. Statistical significance was assessed using a two-way ANOVA with Bonferroni correction for multiple group comparisons, unless otherwise indicated; ns not significant; *p < 0.05, **p < 0.01. Source data are provided in the Source data file.

Next, we investigated the effects of two existing antiviral agents, remdesivir (RDV) and RSV604, on neutrophil-mediated inflammation (timeline shown in Fig. 7A, B), focusing on the production of IL-6, IL-8 and IP-10 and neutrophil marker expression.

Ciliated AECs (Fig. 7C) were infected with GFP-tagged RSV in the presence and absence of RSV604 or RDV. In untreated controls, viral propagation was evident with increasing numbers of GFP-positive cells over a 7-day period, reflecting active infection and spread. In contrast, samples treated with RSV604 showed no detectable RSV-GFP infection throughout the entire time course, indicating that RSV604 effectively inhibits RSV replication and spread in this model (Fig. 7D). Similarly, ALI cultures treated with RDV showed complete absence of GFP expression compared to untreated controls at 7 days post-infection, confirming effective inhibition of RSV replication in this model (Fig. 7E).

Apical supernatants collected before neutrophil introduction revealed a significant reduction in viral load following RDV (1.2 × 10^6^ ± 5.3 × 10^5^ copies/μl) and RSV604 (1.4 × 10^6^ ± 5.0 × 10^5^ copies/μl) treatment compared to untreated controls (3.8 × 10^6^ ± 6.7 × 10^5^ copies/μl, *p* < 0.05, *n* = 6) (Fig. 7F). These measurements, taken 24 h post-infection, should be interpreted with caution since plaque assays at this time point were below the detection limit (data not shown), suggesting that the results may not reflect infectious viral load. Meanwhile, pre-treatment with RDV and RSV604 resulted in a significant increase in IL-6 and IP-10 secretion relative to drug-treated, uninfected controls (Fig. 7G).

### The antiviral RSV604 reduces neutrophil-mediated inflammation during RSV infection

Finally, we aimed to assess whether RDV or RSV604 treatment could specifically reduce the expression of key neutrophil degranulation markers, MPO and NE, which were significantly elevated in both in vitro and in vivo models of RSV infection.

**Fig. 8:**
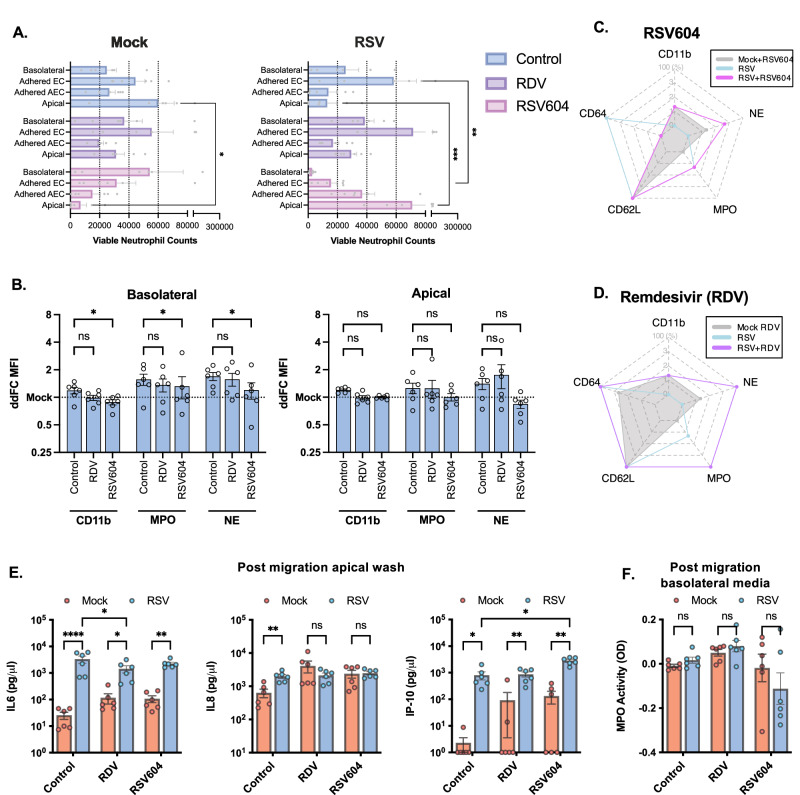
Effects of antiviral treatments on neutrophil degranulation using in vitro RSV models. **A** Total viable neutrophil counts across compartments. No significant differences (ns) in total neutrophil counts across treatments; RSV604 leads to increased migrated neutrophils. Bars represent mean ± SEM (*n* = 6 biologically independent neutrophil samples). Statistical significance was assessed using a two-way ANOVA with Bonferroni correction for multiple group comparisons; *p < 0.05, **p < 0.01**. B** Neutrophil marker expression (ΔΔFC of MFI relative to medium-only and mock controls) across compartments and treatments. Bars represent mean ± SEM (*n* = 6 biologically independent neutrophil samples). Statistical significance was assessed using a two-way ANOVA with Bonferroni correction; *p < 0.05, **p < 0.01**. C**,** D** Radar plots of neutrophil activation marker quartile scores across compartments (basolateral, adhered epithelial, adhered endothelial and migrated neutrophils). RSV604 reduces MPO and NE activation, especially in migrated neutrophils. **E** Post-migration apical cytokine levels of IL-6, IL-8 and IP-10 compared to RSV alone (*p* < 0.05). Bars represent mean ± SEM (*n* = 6 biologically independent neutrophil samples); ns not significant; *p < 0.05, **p < 0.01. Statistical significance was assessed using a two-way ANOVA with Bonferroni correction; *p < 0.05, **p < 0.01**. F** MPO enzymatic activity in basolateral supernatants. RSV alone increases MPO activity (*p* < 0.05 vs mock), while RSV604 and RDV display the same activity levels. Bars represent mean ± SEM (*n* = 6 biologically independent neutrophil samples). Statistical significance was assessed using a two-way ANOVA with Bonferroni correction; *p < 0.05, **p < 0.01. Source data are provided in the Source data file.

Notably, RSV604, but not RDV, led to higher numbers of viable neutrophils recovered from the apical side (Fig. 8A and Supplementary Fig. 8a, b) and was associated with a lower expression of nearly all degranulation markers on the basolateral side (Fig. 8B). The radar plot demonstrates that RSV604 (pink) markedly shifts neutrophil marker expression, particularly NE and MPO, across all compartments, aligning more closely with mock (grey) conditions (Fig. 8C and Supplementary Fig. 8c). This suggests RSV604’s potential to effectively modulate the neutrophil response to RSV infection. In contrast, RDV (purple) shows weaker modulation, with marker expression patterns more closely resembling those of the RSV control (blue) or greater (Fig. 8D).

These changes in neutrophil degranulation marker expression corresponded to minor alterations in inflammatory cytokine levels following neutrophil migration. Neutrophil migration following RSV infection and RDV treatment significantly (*p* < 0.05) reduced IL-6 secretions, while RSV604 led to further increase in IP-10 levels from untreated, infected control (Fig. 8E). Treatment with RSV604 showed a trend towards a reduction in MPO enzymatic activity in the basolateral compartment, consistent with the lower MPO expression observed on basolateral neutrophils, although this reduction did not reach statistical significance (Fig. 8F). These findings highlight the potential of antiviral treatments to attenuate neutrophil degranulation during RSV infection.

## Discussion

In this study, we aimed to develop an in vitro model that recapitulates key clinical outcomes in infants with RSV bronchiolitis to help accelerate the discovery of effective therapeutics. A key finding was that peripheral blood neutrophils from RSV-infected infants had elevated levels of MPO and NE. Interestingly, these infants exhibited a lower overall neutrophil count in circulation, likely reflecting increased recruitment to the lungs, a well-documented feature of RSV bronchiolitis^8,10,20,21^. Elevated MPO and NE levels have also been observed in bronchoalveolar and nasopharyngeal lavage fluid samples from RSV-infected infants^13,14^, and neutrophils recovered from infection sites display activation markers such as increased CD11b and CD64, and reduced CD62L ^22–25^.

To establish our clinically relevant in vitro model, we first investigated whether epithelial age was a principal driver of neutrophil phenotype. Our previous work has shown significant baseline age‑dependent differences in paediatric versus adult airway epithelium^26^, suggesting that epithelial age could be a key determinant of neutrophil behaviour. Here we showed that the paediatric epithelium drives both greater neutrophil migration and a selectively higher MPO and NE signal in basolateral compartment (which models the systemic circulation), closely mirroring the phenotype observed in RSV‑infected infants.

Building on this, we then established a more physiologically relevant airway microenvironment by incorporating a vascular endothelial layer to simulate neutrophil extravasation during RSV infection^27^. As expected for a barrier‑forming compartment, endothelial cells reduced dextran flux but did not alter RSV replication (viral RNA levels) in the epithelium. RSV exposure induced robust production of IL‑6, IL‑8 and IP‑10 across models, consistent previous in vitro studies^11,28^ and with clinical profiles showing elevated IL‑6, IL‑8 and IP‑10 in nasopharyngeal and bronchial aspirates from infants with severe RSV bronchiolitis^29–31^. Interestingly, cytokine levels were lower in the EC‑containing model, suggesting that endothelial cells may modulate early epithelial inflammatory signalling, a finding that contrasts with influenza A virus, where ECs have been shown to amplify epithelial cytokine production ^32^.

Using this model, we found that neutrophil trans-epithelial migration increased markedly following RSV infection, likely driven by elevated IL‑8 and IP‑10. In the absence of endothelial cells, most neutrophils remained adherent to RSV‑infected airway epithelium, whereas the EC‑containing system supported a substantially greater number of fully migrated, detached neutrophils. This is consistent with previous work^11,18^, and suggests that endothelial cells influence neutrophil behaviour, with migrated neutrophils potentially contributing more extensively to RSV‑associated pathology. We found significant differences in basolateral neutrophil expression of MPO and NE, but no other significant differences between RSV and mock conditions were detected. These findings are consistent with the dominant MPO/NE‑driven signature reported previously in an adult in vitro RSV migration system^12^. However, that study showed a stepwise increase in CD11b, NE and MPO with migration, with the highest levels in apical neutrophils. These differences are likely due to differences in epithelial age in the two systems, highlighting the importance of epithelial-neutrophil interactions is determining phenotype. While not the aim of this current study, a logical next step would be to define the adhesion pathways underpinning these age‑dependent effects. Prior work has shown that LFA‑1:ICAM‑1 interactions are essential for neutrophil transepithelial migration in RSV infection^18^, blocking this interaction could help elucidate a mechanistic link between epithelial age and neutrophil response.

Using quartile‑based analyses across all markers, we found that migrated neutrophils displayed a distinct activated phenotype (elevated CD11b, CD64, MPO and NE, with reduced CD62L), mirroring profiles seen in bronchoalveolar lavage fluid and peripheral blood neutrophils from RSV-infected infants^22–25,33,34^. A key finding was that this basolateral neutrophil phenotype appeared to depend on direct contact with RSV-infected epithelial cells rather than soluble factors alone. Notably, basolateral neutrophils that were prevented from migrating did not acquire this phenotype, supporting the hypothesis that trans‑epithelial migration is necessary for full activation. These findings raise the possibility that basolateral neutrophils may re‑enter the circulation after activation, with migration across endothelial and RSV‑infected epithelial layers priming them for degranulation and potentially contributing to systemic inflammation by transporting viral components beyond the lung ^11,35,36^.

Finally, we used this model to investigate the effects of two well-characterised RSV inhibitors, RDV and RSV604, which target distinct stages of RSV replication^37,38^. We found that both significantly reduced viral load and GFP fluorescence in infected AECs, consistent with previous studies^38,39^. However, the reduction in IL-6 levels with RDV, observed only after neutrophil migration, suggests that the presence of neutrophils, possibly in conjunction with reduced viral antigen load, may contribute to a regulatory feedback mechanism that reduces inflammation, thereby improving disease outcomes.

**Fig. 9:**
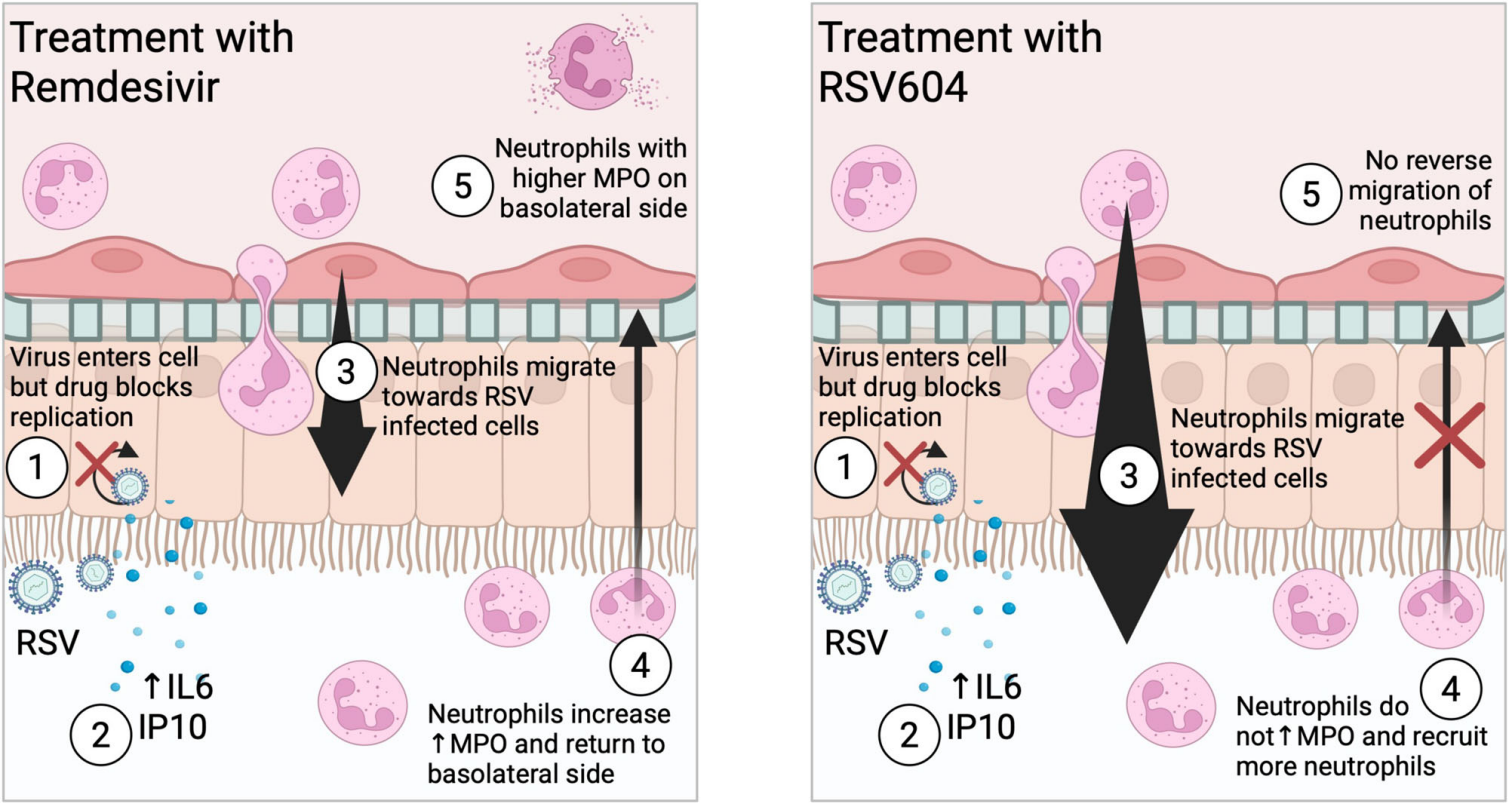
Proposed model of neutrophil migration and activation during RSV infection with and without RSV604 treatment. Schematic diagrams depict epithelial infection, cytokine secretion and neutrophil recruitment under remdesivir (left) and RSV604 (right) treatment conditions. In the remdesivir model, infected epithelial cells release IL‑6 and IP‑10, leading to neutrophil activation, increased MPO and NE expression, and basolateral migration with elevated degranulation. In contrast, RSV604 reduces epithelial cell infection and inflammatory signalling, resulting in neutrophil migration with lower CD11b, MPO and NE expression. Together, these diagrams illustrate a potential mechanism by which RSV604 promotes the recruitment of less‑activated neutrophils and limits tissue‑damaging inflammation. Created in BioRender. Woodall, M. (2026) https://BioRender.com/gt08btt.

Interestingly, RSV604 increased the number of viable neutrophils migrating across infected co-cultures. Neutrophils play a crucial role in viral clearance, but excessive recruitment and degranulation can cause tissue damage. However, our data indicate that neutrophils migrating under RSV604 treatment were less activated, with reduced expression of CD11b, MPO and NE. This suggests that RSV604 may promote the recruitment of less activated neutrophils, which could mitigate excessive inflammation. Further research is needed to explore how RSV604 influences these pathways and whether it has the potential to modulate neutrophils in vivo. A working hypothesis is shown in Fig. 9.

Limitations of this study include the use of paediatric neutrophils for in vivo analysis and adult neutrophils for in vitro experiments. This was principally due to logistical challenges in obtaining healthy paediatric blood samples on the morning of the migration assay. Although direct comparison between healthy paediatric and adult donors revealed only differences in the resting state of CD64, other age-related differences in neutrophil responsiveness or priming potential may still influence the observed outcomes. Additionally, the in vitro model, while physiologically relevant, does not fully recapitulate the complexity of the in vivo airway environment, including interactions with other immune cell types and systemic factors.

To conclude, our study identifies myeloperoxidase (MPO) as a key marker of neutrophil activation and a potential systemic indicator of disease severity in RSV infection. Using a clinically informed in vitro model, we showed that neutrophil activation is primarily driven by direct contact with RSV-infected epithelial cells, resulting in increased expression of CD11b, MPO and NE, features associated with severe disease. MPO emerged as the most significantly modulated marker, confirmed by both expression and enzymatic activity assays. Although both RDV and RSV604 reduced MPO levels compared to RSV alone, neither restored them to baseline, indicating only partial suppression of degranulation. Notably, only RSV604 effectively reduced MPO expression, suggesting its potential to mitigate neutrophil-mediated tissue damage. Furthermore, high MPO expression in basolateral neutrophils points to a possible role in systemic inflammation. These results suggest that neutrophil MPO expression should be considered a relevant biomarker in the evaluation and screening of antiviral therapies for RSV.

## Method

### Ethical approval and consent

All studies involving human participants were conducted in accordance with the Declaration of Helsinki. Studies involving paediatric patients admitted to the Great Ormond Street Hospital paediatric intensive care unit (PICU) was approved through the Living Airway Biobank, administered through the University College London (UCL) Great Ormond Street Institute of Child Health (REC reference: 19/NW/0171, IRAS project ID: 261511, Northwest Liverpool East Research Ethics Committee). Ethical approval for the collection and use of venous blood from healthy adult volunteers was obtained from the UCL Research Ethics Committee (Project ID: 19165/001). Written informed consent was obtained from the parents or legal guardians of all infant participants, and from all adult donors prior to sample collection.

Primary airway epithelial cells (AECs) from adult donors were obtained as previously described^12^. Paediatric primary airway epithelial cells were either obtained via the Living Airway Biobank (as above) or purchased from Epithelix Sàrl (EP61SA, Geneva, Switzerland). Commercially sourced paediatric bronchial AECs were supplied fully de‑identified, collected under Epithelix Sàrl review board-approved protocols with written informed consent; their use in this study therefore did not require additional ethical approval. All methods were performed following the relevant guidelines and regulations.

### Neutrophil isolation and purification

Neutrophils were isolated from children in PICU with RSV infection with an indwelling arterial or central venous line (*n* = 5, age range 1.5–29 months, 60% female) and uninfected control PICU patients (who have had elective procedures) (*n* = 5, age range 0.2–7 months, 80% female) (Figs. 1C and 2B; detailed patient characteristics in Table 1) or healthy adults (*n* = 15, aged 20–40 years, 58% female) (patient characteristics in Table 1). Blood sampling was carried out by trained phlebotomists. Neutrophils were isolated using the EasySep Direct Human Neutrophil Isolation Kit (#19666, STEMCELL Technologies, UK), yielding approximately 1 × 10^7^ neutrophils with up to 99% purity. Briefly, the sample was incubated with 500 µl of the Isolation Cocktail and RapidSpheres, followed by a magnetic separation in 50 ml of cell separation buffer. The clear fraction was collected, re-incubated with RapidSpheres, and placed on a magnetic stand. After further incubation and centrifugation at 300 × *g*, the pellet was resuspended in separation buffer and further enriched using the EasySep Human Neutrophil Isolation Kit (#17957, STEMCELL Technologies, UK) as per the manufacturer’s instructions.

### Airway epithelial cell culture and differentiation

Primary airway epithelial cells (AECs) were obtained from adults (*n* = 6, 19–40 years as described in ref.^12^.) and children (*n* = 5, 0–4 years) by nasal brushings. Primary paediatric (2-year-old female Caucasian) bronchial AECs were purchased from Epithelix Sàrl (EP61SA, Geneva, Switzerland) and co-cultured on mitotically inactivated 3T3-J2 feeder cells as described previously^40,41^. Cell stocks were frozen down at 1 × 10^6^ cells/100 µl in Bambanker (#K1-0660, NIPPON Genetics, Germany), a serum-free cryopreservation medium. AECs were then cultured at ALI in complete PneumaCult-ALI medium (#05001, STEMCELL Technologies, Vancouver, Canada) for 28 days to promote differentiation as described previously ^27^.

### Endothelial cell culture

Human umbilical vein endothelial cells (HUVECs) transduced with ETS variant transcription factor 2, also known as ‘reset’ vascular ECs^42^, were cultured on recombinant laminin-511 E8 fragment-coated flasks (iMatrix-511, #AMS.892 012, AMSBIO, UK) in endothelial growth medium composed of complete Endopan 300 SL medium (#P04-0065K, Pan BioTech, Germany) and 5% human serum (#H3667, Merck, Darmstadt, Germany). These reagents proved to be the most effective growth conditions for endothelial cells of others tested (Supplementary Fig. 2). Cell stocks were frozen down at 1 × 10^6^ cells/ml in EGM supplemented with 40% HS and 10% DMSO.

For co-culture at ALI, ECs were seeded into membrane inserts at 3.5 × 10^4^ in 100 μl complete PneumaCult-ALI medium supplemented with iMatrix-511 (1.5 μg/cm^2^) and incubated for 24 h prior to performing dextran permeability assays or neutrophil migration experiments.

### Dextran permeability assay

To assess cell barrier integrity, membrane inserts with ALI AEC cultures alone or in co-culture with ECs, were placed in Hanks’ balanced salt solution containing calcium and magnesium (HBSS+/+) plus Texas Red-dextran (#D3328, Thermo Fisher Scientific, UK) at 100 µg/ml for 20 min in the dark. One hundred microliters of standards and supernatant were transferred to a solid black 96-well plate in triplicate, and fluorescence was measured with a BMG FLUOstar OMEGA microplate reader at an excitation/emission wavelength of 595/615 nm. A membrane without cells served as a control. The translocated dextran concentration, derived from a standard curve, indicated paracellular permeability.

### Viral infection and quantification

Recombinant GFP tagged-RSV A2 strain was provided by Fix et al^43^. Virus stocks were propagated in HEp-2 (Human Epithelial type 2) cells (#CCL23, ATCC) as previously described^44^. ALI cultures were infected at a MOI of 1 in 25 µl HBSS+/+ for 1 h at 37 °C. The virus quantity was based on an estimated 1 × 10^5^ cells per membrane. Mock-infected controls received 25 µl HBSS+/+. After inoculation, the virus solution was removed, and cultures were incubated for 24 h with replenished basolateral ALI medium.

For quantification, viral RNA was extracted from apical supernatants using the QIAamp Viral RNA Mini Kit (#52906, Qiagen, Germany). cDNA was synthesised from 100 ng RNA using the High-Capacity RNA-to-cDNA Kit (#4387406, Thermo Fisher Scientific, UK) in a 40 μl reaction. RT-qPCR was performed using TaqMan Universal Master Mix II (#4440042, Thermo Fisher Scientific, UK) with specific primers and a fluorescent probe for the RSV-A N protein as described previously^27^. Oligonucleotide sequences are provided in Supplementary Table 1.

Samples were run on an AB Biosystems StepOnePlus Real-Time PCR System (Thermo Fisher Scientific, UK). Viral load was extrapolated from a standard curve generated by performing ten-fold serial dilutions of a plasmid containing the N protein sequence, starting at 1 × 10^6^ copies to 1 copy ^45^.

### Trans-epithelial migration assay

On the day of the neutrophil migration assay, ALI cultures were incubated with 600 µl HBSS+/+ for 1 h at 37 °C to collect immunomodulatory factors. Supernatants were collected, centrifuged, and 400 µl was transferred to a 24-well plate with ALI cultures. Purified neutrophils were diluted to 5 × 10^6^ cells/ml in HBSS+/+ with 1% (v/v) autologous human serum. One hundred microliters was added basolaterally to each membrane insert and incubated for 1 h at 37 °C.

### Assessment of neutrophil markers and degranulation

After migration, neutrophils (apical, adherent and basolateral) were collected, centrifuged, blocked with Human TruStain FcX (#422302, BioLegend, USA), and stained with LIVE/DEAD fixable violet dye (#L34963, Thermo Fisher Scientific, UK). Following washing, cells were stained with 1/50 dilution of CD11b-FITC (#130-110-552, Miltenyi Biotec), CD64-APC-Cy7 (#130-116-199, Miltenyi Biotec), and CD62L-PE-Cy7 (#130-129-810, Miltenyi Biotec) for 20 min at 4 °C in the dark. Cells were washed, fixed with PFA, permeabilised and stained intracellularly with 1/50 dilution of MPO-APC (#130-119-786, Miltenyi Biotec) and NE-PE (#sc-55549 PE, Santa Cruz Biotechnology). After a final wash, neutrophils were resuspended in FACS buffer and analysed using a CytoFLEX S flow cytometer, obtaining mean fluorescence intensity (MFI) values for CD11b, CD64, CD62L, MPO and NE from the CD11b-positive gated population. The gating strategy for the identification of neutrophils by flow cytometry is shown in Supplementary Fig. 1. Dilutions and catalogue numbers for all antibodies are provided in Supplementary Table 2. Data are presented as raw MFI values, or to control for day-to-day variability, we normalised MFI from migration experiments to same-day 'medium-only' controls and use FMO and cytometer calibration/PMT stability checks. To visualise patterns of marker expression, we utilised a Quartile Score, where the mean MFI of each marker was categorised into quartiles (0–3); 0 corresponds to the lowest quartile and 3 to the highest, calculated across all compartments and experimental conditions. Associations between marker expression patterns across different compartments or conditions of the model were visualised using radar charts generated with the fmsb R package^46^. Here, the centre of each plot represents the lowest level of marker expression, while points closer to the edges indicate higher expression levels. The individual quartile scores were totalled to calculate a combined quartile score for each condition. Statistical analyses were performed using Wilcoxon signed-rank tests, with adjustments for multiple comparisons to control the false discovery rate.

Neutrophil elastase activity in supernatants was measured using the fluorometric Neutrophil Elastase Activity Assay Kit (#600610, Cayman Chemical, Michigan, USA) as per the manufacturer’s instructions. Samples were diluted 1/10, incubated with elastase substrate, and fluorescence was measured at 485/525 nm with a BMG FLUOstar OMEGA microplate reader.

For MPO activity, samples were diluted 1/2 and incubated with 3,3',5,5'-tetramethylbenzidine substrate as described using the colorimetric Neutrophil MPO Activity Assay Kit (#600620 Cayman Chemical, Michigan, USA). Absorbance was measured at 650 nm with a BMG FLUOstar OMEGA microplate reader.

### Transcriptomics

Neutrophil RNA from RSV-infected and control paediatric patients was extracted using the RNeasy Plus Mini Kit (Qiagen, Germany). RNA samples were then reverse-transcribed and sequenced using the cDNA-PCR Sequencing V14 - Barcoding kit (SQK-PCB114.24) according to the manufacturer’s instructions. Samples were sequenced on the R10.4.1 flow cell with a MinION Mk 1C sequencer (Oxford Nanopore Technologies, UK). Raw sequencing data were base-called during acquisition via MinKNOW (Oxford Nanopore Technologies). Reads were processed using the ONT EPI2ME: wf-transcriptomes workflow to generate aligned BAM files against the human reference genome GRCh38.p14.115. Gene-level counts were subsequently quantified from the aligned BAM files using featureCounts (Subread package), generating a raw gene count matrix for downstream analysis. Differential gene expression between RSV and control groups was conducted using the edgeR package. Lowly expresses genes were filtered by retaining genes with CPM ≥ 1 in at least two samples. Library size normalisation was performed using the trimmed mean of *M* values (TMM) method implemented in edgeR. Principal component (PC) analysis was performed on log2-transformed normalised expression values (log2(CPM + 1) using the top 500 most variable genes. To visualise genes contributing to PC2 separation, the top 250 genes with the highest positive and 250 genes with the highest negative PC2 loadings were selected corresponding to RSV-enriched and Control-enriched gene sets, respectively, scaled (z-scored per gene) and visualised using hierarchical clustering heatmaps. Gene ontology (GO) enrichment analysis was performed using Enrichr through GSEApy Python interface, focusing on GO biological process 2021 annotations. Enrichment significance was evaluated using adjusted *P*-values (Benjamini–Hochberg correction), and enrichment strength was quantified using the Enrichr combined score. Shared GO terms between RSV-up and control-up gene sets were identified.

### Cytokine measurement

The BD cytometric bead array (CBA) assay (#558264, BD Biosciences, USA) was used to quantify human soluble proteins, including key mediators of inflammation and neutrophil migration, IL-6, CXCL8 (IL-8) and CXCL10 (IP-10)^47,48^, in supernatants from mock- or RSV-infected cultures. Following the manufacturer’s protocol, a standard curve was generated, and test samples were diluted 1/10 in assay diluent. Standards and samples were incubated with capture beads for 1 h, followed by PE detection reagent for 2 h. After washing, the beads were resuspended in wash buffer and analysed using a BD FACSymphony A5 flow cytometer (BD Biosciences, Franklin Lakes, NJ, USA).

### Antiviral treatment

RSV-infected bronchial AECs were treated with a broad-spectrum antiviral remdesivir (RDV, #7226, Bio-Techne, USA) or RSV-specific antiviral compound, RSV604 (#HY-12993, Cambridge Bioscience, UK), which has high efficacy in inhibiting RSV replication^38^. RDV and RSV604 were diluted in ALI medium to working concentrations of 6.6 and 10 μM, respectively, and added to the basolateral side at 4 h post-infection. These concentrations were based on RDV EC50 values^49^ and previously reported non-cytotoxic RSV604 doses effective against RSV in human AEC^38^. AECs were used for neutrophil migration experiments at 1 day post-infection (dpi). The effects of the antiviral on RSV-induced neutrophil migration and receptor expression were assessed by comparing treated and untreated cultures. Additional infected AECs with and without antiviral treatment were recorded for viral GFP fluorescence using a Nikon Ti-E microscope up to 7 dpi.

### Statistical analyses

Data were analysed and visualised using GraphPad Prism v10.0 or RStudio 2023.09.1 + 494. It is important to note that there was considerable variability (>1 log) in baseline neutrophil marker expression across different donors (Supplementary Fig. 3). This variability likely reflects inherent differences in neutrophil states between individuals at collection. To account for this in our analysis, we present our data as the mean fold change in MFI relative to neutrophils cultured only in medium (‘medium-only’) for each condition. This approach normalised the individual baseline differences and allowed us to focus on the migration-dependent effects.

Normality was assessed with the Shapiro-Wilk test. For normally distributed data, a Student’s *t* test (two groups) or one-way ANOVA with Bonferroni correction (three or more groups) was used. Two-way ANOVA with Tukey correction was applied for comparisons involving two factors. Non-normally distributed data were analysed with Mann–Whitney *U* or Wilcoxon signed-rank tests (two groups) and Kruskal–Wallis or Friedman tests with Dunn’s post-hoc for unpaired or paired data, respectively. Results are presented as mean ± SEM unless specified otherwise. Statistical significance was set at *P* < 0.05.

For age-adjusted analyses, data were log‑transformed and analysed in R using linear and logistic regression to assess effects of RSV status and age, with effect ratios, odds ratios and predicted values derived using broom, questionr and ggeffects.

### Reporting summary

Further information on research design is available in the Nature Portfolio Reporting Summary linked to this article.

## Supplementary information

Supplementary Information

Description of Additional Supplementary Files

Supplementary Video 1

Supplementary Video 2

Supplementary Video 3

Reporting Summary

Transparent Peer Review File

## Source data

Source Data

## Data Availability

RNA-seq data generated in this study have been deposited in the European Genome-Phenome Archive (EGA) under accession code EGAD50000002668 https://ega-archive.org/. To access the data, a Data Access Request (DAR) needs to be submitted on the EGA dataset page, which will be further viewed by the corresponding author. These RNA‑seq data are available under controlled access due to the inclusion of human participant data and associated privacy regulations. Access can be obtained by submitting a data‑access request to the corresponding EGA Data Access Committee. Processed RNA‑seq data and all other data supporting the conclusions of this study are provided within the article, Supplementary Information or the accompanying Source data file. Source data are provided with this paper.

## Code availability

Custom code for the analysis performed in this study is publicly available via GitHub at https://github.com/smithlab-code/NCOMMS-25-82134.git.

## References

[1] Shi, T. et al. Global, regional, and national disease burden estimates of acute lower respiratory infections due to respiratory syncytial virus in young children in 2015: a systematic review and modelling study. *Lancet* **390**, 946–958 (2017). 28689664 10.1016/S0140-6736(17)30938-8 PMC5592248

[2] Frankel, L. R. et al. A technique for the administration of ribavirin to mechanically ventilated infants with severe respiratory syncytial virus infection. *Crit. Care Med.* **15**, 1051–1054 (1987). 3677748 10.1097/00003246-198711000-00012

[3] Alvarez de Toledo, M., Taveras, J. I., Ramilo, O. & Mejias, A. Antiviral therapies for respiratory syncytial virus infection: current expectations. *Curr. Opin. Infect. Dis.* **39**, 153–159 (2026). 41593430 10.1097/QCO.0000000000001184

[4] American Academy of Pediatrics Committee on Infectious Diseases & American Academy of Pediatrics Bronchiolitis Guidelines Committee. Updated guidance for palivizumab prophylaxis among infants and young children at increased risk of hospitalization for respiratory syncytial virus infection. *Pediatrics* **134**, 415–420 (2014). 10.1542/peds.2014-1665 25070315

[5] Subramanian, K. N. et al. Safety, tolerance and pharmacokinetics of a humanized monoclonal antibody to respiratory syncytial virus in premature infants and infants with bronchopulmonary dysplasia. MEDI-493 study group. *Pediatr. Infect. Dis. J.* **17**, 110–115 (1998). 9493805 10.1097/00006454-199802000-00006

[6] Griffin, M. P. et al. Single-dose nirsevimab for prevention of RSV in preterm infants. *New Engl. J. Med.* **383**, 415–425 (2020). 32726528 10.1056/NEJMoa1913556

[7] Hammitt, L. L. et al. Nirsevimab for prevention of RSV in healthy late-preterm and term infants. *New Engl. J. Med.* **386**, 837–846 (2022). 35235726 10.1056/NEJMoa2110275

[8] McNamara, P. S., Ritson, P., Selby, A., Hart, C. A. & Smyth, R. L. Bronchoalveolar lavage cellularity in infants with severe respiratory syncytial virus bronchiolitis. *Arch. Dis. Child.* **88**, 922–926 (2003). 14500316 10.1136/adc.88.10.922 PMC1719332

[9] Wang, S. Z. et al. Neutrophils induce damage to respiratory epithelial cells infected with respiratory syncytial virus. *Eur. Respir. J.* **12**, 612–618 (1998). 9762789 10.1183/09031936.98.12030612

[10] Kirsebom, F. C. M., Kausar, F., Nuriev, R., Makris, S. & Johansson, C. Neutrophil recruitment and activation are differentially dependent on MyD88/TRIF and MAVS signaling during RSV infection. *Mucosal Immunol.* **12**, 1244–1255 (2019). 31358860 10.1038/s41385-019-0190-0 PMC6778055

[11] Robinson, E. et al. Trans-epithelial migration is essential for neutrophil activation during RSV infection. *J. Leukoc. Biol.* **113**, 354–364 (2023). 36807711 10.1093/jleuko/qiad011 PMC11334017

[12] Robinson, E. et al. Neutrophil responses to RSV infection show differences between infant and adult neutrophils. *Thorax* **79**, 545–552 (2024). 38050163 10.1136/thorax-2023-220081 PMC11137455

[13] Emboriadou, M. et al. Human neutrophil elastase in RSV bronchiolitis. *Ann. Clin. Lab. Sci.* **37**, 79–84 (2007). 17311874

[14] Sigurs, N., Bjarnason, R. & Sigurbergsson, F. Eosinophil cationic protein in nasal secretion and in serum and myeloperoxidase in serum in respiratory syncytial virus bronchiolitis: relation to asthma and atopy. *Acta Paediatr.* **83**, 1151–1155 (1994). 7841728 10.1111/j.1651-2227.1994.tb18269.x

[15] Halfhide, C. P. et al. Respiratory syncytial virus binds and undergoes transcription in neutrophils from the blood and airways of infants with severe bronchiolitis. *J. Infect. Dis.* **204**, 451–458 (2011). 21742845 10.1093/infdis/jir280 PMC3132143

[16] Zemans, R. L., Colgan, S. P. & Downey, G. P. Transepithelial migration of neutrophils: mechanisms and implications for acute lung injury. *Am. J. Respir. Cell Mol. Biol.* **40**, 519–535 (2009). 18978300 10.1165/rcmb.2008-0348TR PMC2677434

[17] Taylor, G. Animal models of respiratory syncytial virus infection. *Vaccine* **35**, 469–480 (2017). 27908639 10.1016/j.vaccine.2016.11.054 PMC5244256

[18] Herbert, J. A. et al. Beta-integrin LFA1 mediates airway damage following neutrophil transepithelial migration during respiratory syncytial virus infection. *Eur. Respir. J.* **56**, 1902216 (2020). 10.1183/13993003.02216-2019 PMC7406857 32217648

[19] Everard, M. L. et al. Analysis of cells obtained by bronchial lavage of infants with respiratory syncytial virus infection. *Arch. Dis. Child.* **71**, 428–432 (1994). 7826113 10.1136/adc.71.5.428 PMC1030058

[20] Kirsebom, F., Michalaki, C., Agueda-Oyarzabal, M. & Johansson, C. Neutrophils do not impact viral load or the peak of disease severity during RSV infection. *Sci. Rep.* **10**, 1110 (2020). 31980667 10.1038/s41598-020-57969-w PMC6981203

[21] O’Donnell, D. R., McGarvey, M. J., Tully, J. M., Balfour-Lynn, I. M. & Openshaw, P. J. Respiratory syncytial virus RNA in cells from the peripheral blood during acute infection. *J. Pediatr.* **133**, 272–274 (1998). 9709720 10.1016/s0022-3476(98)70234-3

[22] Cassatella, M. A., Ostberg, N. K., Tamassia, N. & Soehnlein, O. Biological roles of neutrophil-derived granule proteins and cytokines. *Trends Immunol.* **40**, 648–664 (2019). 31155315 10.1016/j.it.2019.05.003

[23] Hoffmann, J. J. Neutrophil CD64: a diagnostic marker for infection and sepsis. *Clin. Chem. Lab. Med.* **47**, 903–916 (2009). 19642859 10.1515/CCLM.2009.224

[24] Simon, S. I. et al. L-selectin (CD62L) cross-linking signals neutrophil adhesive functions via the Mac-1 (CD11b/CD18) beta 2-integrin. *J. Immunol.* **155**, 1502–1514 (1995). 7543524

[25] Weirich, E. et al. Neutrophil CD11b expression as a diagnostic marker for early-onset neonatal infection. *J. Pediatr.* **132**, 445–451 (1998). 9544899 10.1016/s0022-3476(98)70018-6

[26] Woodall, M. N. J. et al. Age-specific nasal epithelial responses to SARS-CoV-2 infection. *Nat. Microbiol.* **9**, 1293–1311 (2024). 38622380 10.1038/s41564-024-01658-1 PMC11087271

[27] Palor, M. et al. Ciliated epithelial cell differentiation at air-liquid interface and RSV infection using animal-free media and substrates. *ERJ Open Res.* **11**, 00028–2025 (2025). 41367661 10.1183/23120541.00028-2025 PMC12683564

[28] Villenave, R. et al. Differential cytopathogenesis of respiratory syncytial virus prototypic and clinical isolates in primary pediatric bronchial epithelial cells. *Virol. J.* **8**, 43 (2011). 21272337 10.1186/1743-422X-8-43 PMC3039598

[29] Tabarani, C. M. et al. Novel inflammatory markers, clinical risk factors and virus type associated with severe respiratory syncytial virus infection. *Pediatr. Infect. Dis. J.* **32**, e437–e442 (2013). 23804121 10.1097/INF.0b013e3182a14407 PMC3883981

[30] Diaz, P. V. et al. Levels of inflammatory cytokines and plasma cortisol in respiratory syncytial virus bronchiolitis. *Rev. Med. Chil.* **141**, 574–581 (2013). 24089271 10.4067/S0034-98872013000500004

[31] McNamara, P. S., Flanagan, B. F., Hart, C. A. & Smyth, R. L. Production of chemokines in the lungs of infants with severe respiratory syncytial virus bronchiolitis. *J. Infect. Dis.* **191**, 1225–1232 (2005). 15776367 10.1086/428855

[32] Bauer, L. et al. The pro-inflammatory response to influenza A virus infection is fueled by endothelial cells. *Life Sci Alliance* **6**, e202201837 (2023). 10.26508/lsa.202201837 PMC10114347 37072183

[33] Cowland, J. B. & Borregaard, N. Granulopoiesis and granules of human neutrophils. *Immunol. Rev.* **273**, 11–28 (2016). 27558325 10.1111/imr.12440

[34] Halfhide, C. P. et al. Neutrophil TLR4 expression is reduced in the airways of infants with severe bronchiolitis. *Thorax* **64**, 798–805 (2009). 19497921 10.1136/thx.2008.107821 PMC2730558

[35] Nourshargh, S., Renshaw, S. A. & Imhof, B. A. Reverse migration of neutrophils: where, when, how, and why? *Trends Immunol.* **37**, 273–286 (2016). 27055913 10.1016/j.it.2016.03.006

[36] Buckley, C. D. et al. Identification of a phenotypically and functionally distinct population of long-lived neutrophils in a model of reverse endothelial migration. *J. Leukoc. Biol.* **79**, 303–311 (2006). 16330528 10.1189/jlb.0905496

[37] Frediansyah, A., Nainu, F., Dhama, K., Mudatsir, M. & Harapan, H. Remdesivir and its antiviral activity against COVID-19: A systematic review. *Clin. Epidemiol. Glob. Health* **9**, 123–127 (2021). 32838064 10.1016/j.cegh.2020.07.011 PMC7410793

[38] Chapman, J. et al. RSV604, a novel inhibitor of respiratory syncytial virus replication. *Antimicrob. Agents Chemother.* **51**, 3346–3353 (2007). 17576833 10.1128/AAC.00211-07 PMC2043207

[39] Warren, T. K. et al. Therapeutic efficacy of the small molecule GS-5734 against Ebola virus in rhesus monkeys. *Nature* **531**, 381–385 (2016). 26934220 10.1038/nature17180 PMC5551389

[40] Butler, C. R. et al. Rapid expansion of human epithelial stem cells suitable for airway tissue engineering. *Am. J. Respir. Crit. Care Med.* **194**, 156–168 (2016). 26840431 10.1164/rccm.201507-1414OC PMC5003214

[41] Hynds, R. E., Butler, C. R., Janes, S. M. & Giangreco, A. Expansion of human airway basal stem cells and their differentiation as 3D tracheospheres. *Methods Mol. Biol.* **1576**, 43–53 (2019). 27539459 10.1007/7651_2016_5

[42] Palikuqi, B. et al. Adaptable haemodynamic endothelial cells for organogenesis and tumorigenesis. *Nature* **585**, 426–432 (2020). 32908310 10.1038/s41586-020-2712-z PMC7480005

[43] Fix, J., Galloux, M., Blondot, M. L. & Eleouet, J. F. The insertion of fluorescent proteins in a variable region of respiratory syncytial virus L polymerase results in fluorescent and functional enzymes but with reduced activities. *Open Virol. J.* **5**, 103–108 (2011). 21966341 10.2174/1874357901105010103 PMC3178903

[44] Palor, M. et al. Ciliated epithelial cell differentiation at air–liquid interface and respiratory syncytial virus infection using animal-free media and substrates. *ERJ Open Res.* **11**, 00028-2025 (2025). 41367661 10.1183/23120541.00028-2025 PMC12683564

[45] Castagne, N. et al. Biochemical characterization of the respiratory syncytial virus P-P and P-N protein complexes and localization of the P protein oligomerization domain. *J. Gen. Virol.* **85**, 1643–1653 (2004). 15166449 10.1099/vir.0.79830-0

[46] Nakazawa, M. fmsb: Functions for Medical Statistics Book with some Demographic Data. R package version 0.7.6. (CRAN, 2024).

[47] Haeberle, H. A. et al. Respiratory syncytial virus-induced activation of nuclear factor-kappaB in the lung involves alveolar macrophages and toll-like receptor 4-dependent pathways. *J. Infect. Dis.* **186**, 1199–1206 (2002). 12402188 10.1086/344644

[48] Ouyang, Y. et al. Innate immune evasion by human respiratory syncytial virus. *Front. Microbiol.* **13**, 865592 (2022). 35308390 10.3389/fmicb.2022.865592 PMC8931408

[49] Ellis, S. et al. Polymerase-inhibitor drug synergy and mutational signatures in different epithelial cell models of RSVA and hPIV3 infection. npj Antimicrob Resist (2026). 10.1038/s44259-026-00222-7. 10.1038/s44259-026-00222-7 PMC13530243 42215780

