# Supplementary material for: Neutrophil myeloperoxidase as a functional biomarker for RSV severity: implications for in vitro therapeutic screening
Source: Nat Commun. 2026 Jul 13;17:5507. doi: 10.1038/s41467-026-74414-0 (PMC13365213; doi:10.1038/s41467-026-74414-0)
Supplement: Supplementary file 1 — Supplementary Information [file 41467_2026_74414_MOESM1_ESM.pdf]

Supplementary material for:

Palor M, Masonou T, Robinson EJ, Chen W, Ellis S, Buggiotti L, Jacobs AI, Benoist T, De Coppi P, Rohn JL, Pollara G, Cortina-Borja M, Woodall MNJ, Hynds RE, Smyth RL, Ray S, Smith CM. (2026). Neutrophil myeloperoxidase as a functional biomarker for RSV severity: implications for *in vitro* therapeutic screening.

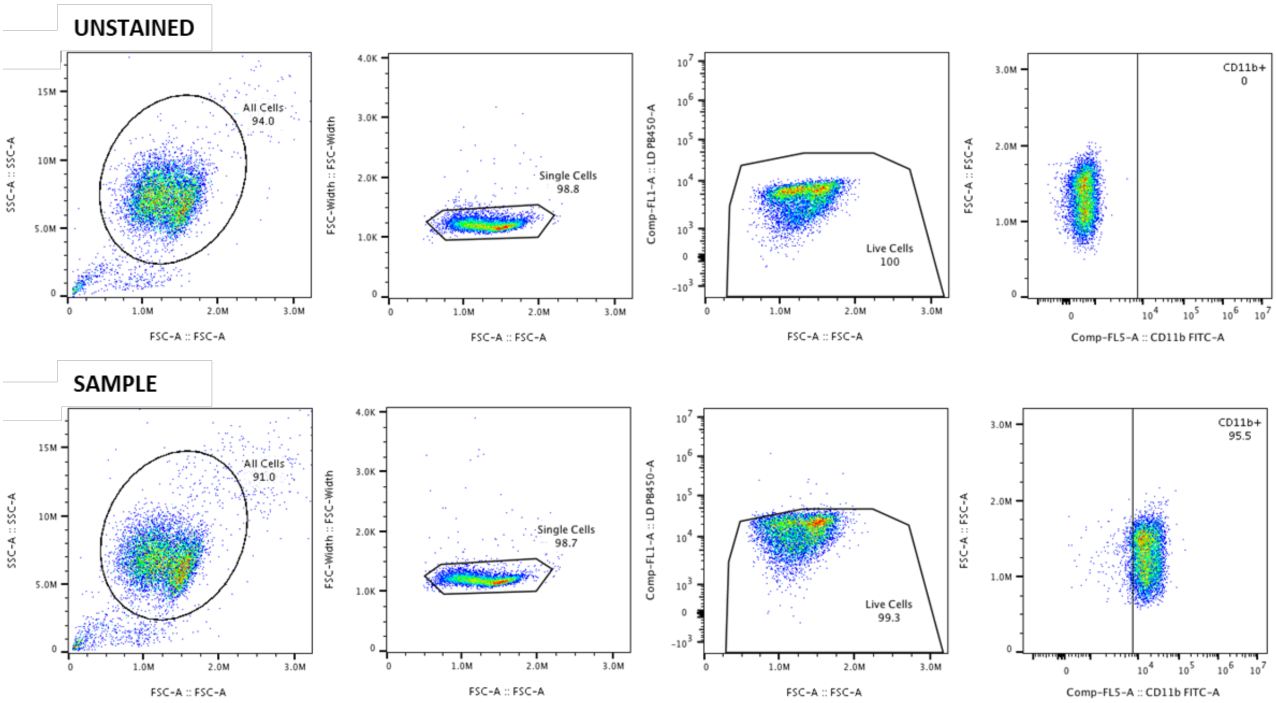

Supplementary Figure 1. Gating strategy to determine expression of neutrophil markers. Neutrophils were defined as the proportion of CD11b-positive cells from the live cell population. MFI values of CD11b, CD64, CD62L, MPO and NE were obtained from the CD11b-positive gated population.

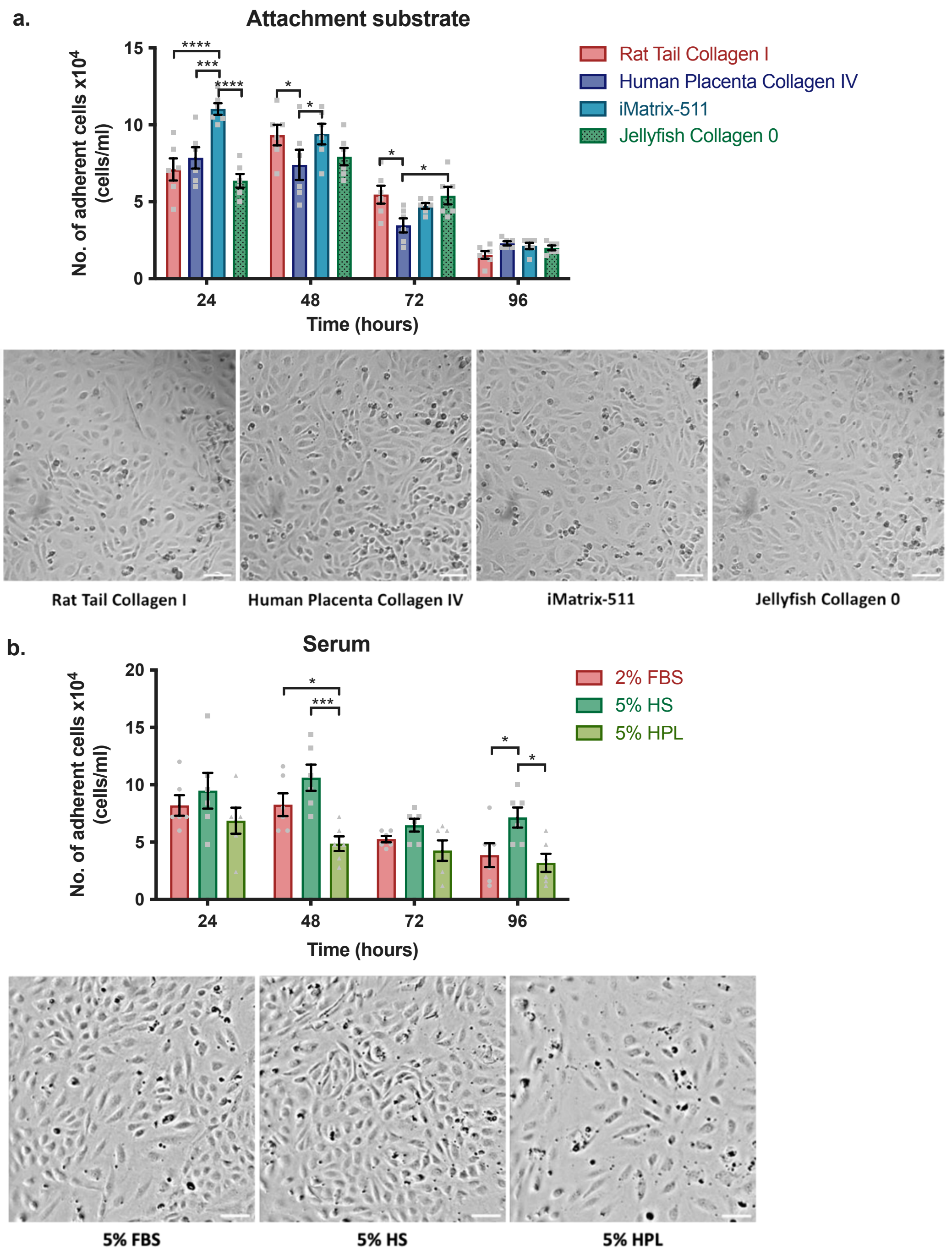

Supplementary Figure 2. Impact of Attachment Substrate and Serum on Endothelial Cell Culture. (a) Top: Comparison of cell attachment across different substrate conditions. Bars represent mean ± SEM of n = 6 independent experiments. *p<0.05, ***p < 0.001, ****p < 0.0001 (Two-Way ANOVA with Tukey post-hoc test). Bottom: representative phase-contrast images at 24 hours. Scale bars = 100 μm. (b) Top number of adherent R-VECs at 24, 48, 72 and 96 hours. Bars represent mean ± SEM of n = 6 independent experiments. *p < 0.05, ***p < 0.001 (Two-Way ANOVA with Tukey posthoc test). Bottom: representative phase-contrast images at 24 hours. Scale bars = 100 μm.

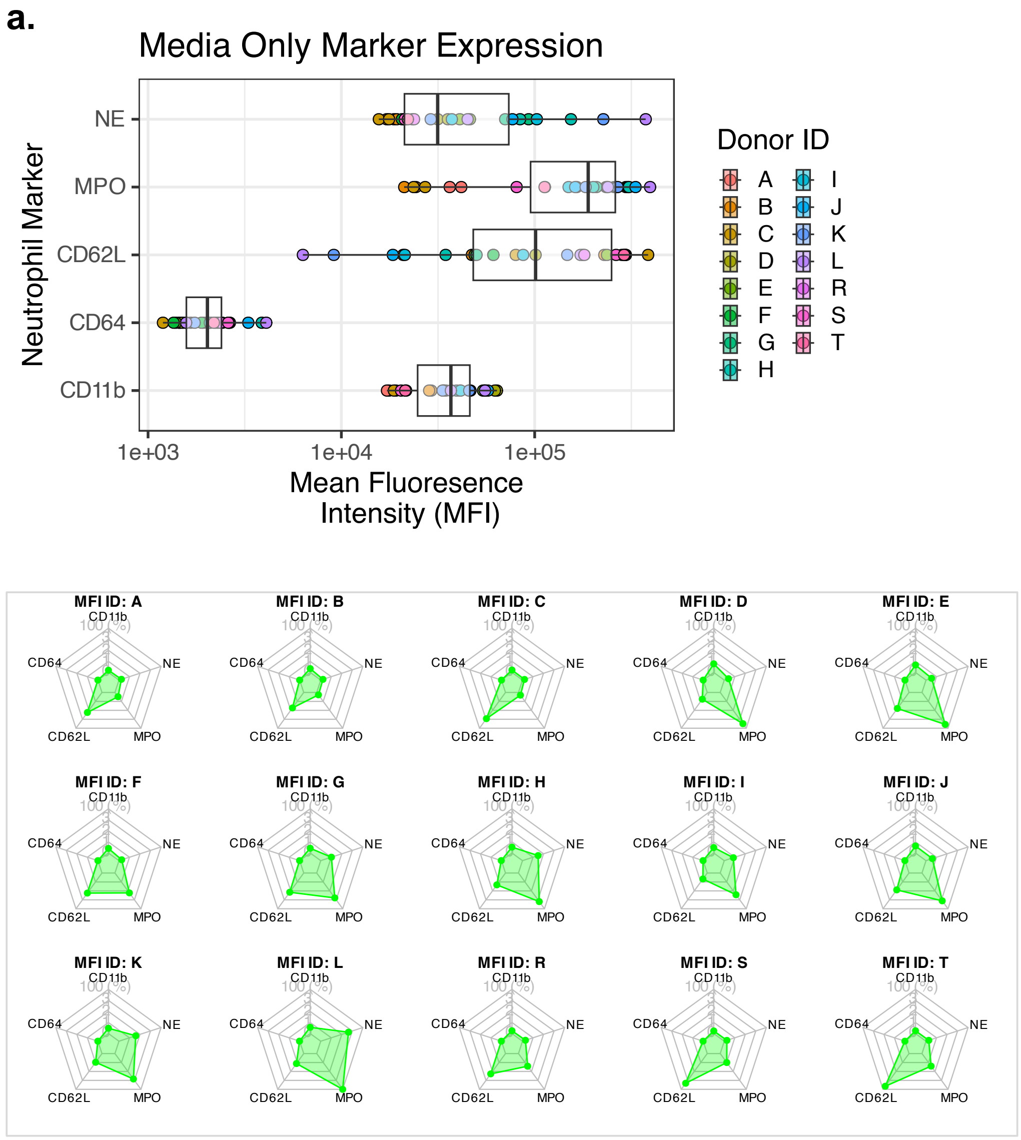

Supplementary Figure 3. Donor Variability in Neutrophil Marker Expression and Cytokine Production in Response to RSV. (a) Mean fluorescence intensity (MFI) of neutrophil markers (NE, MPO, CD62L, CD64, CD11b) for individual donors under media-only conditions, demonstrating substantial inter-donor variation. Boxplots display median and interquartile range (n=15 biologically independent neutrophil samples) (b) Radar plots displaying donor-specific neutrophil activation profiles (donor IDs A–T), highlighting heterogeneity in marker expression.

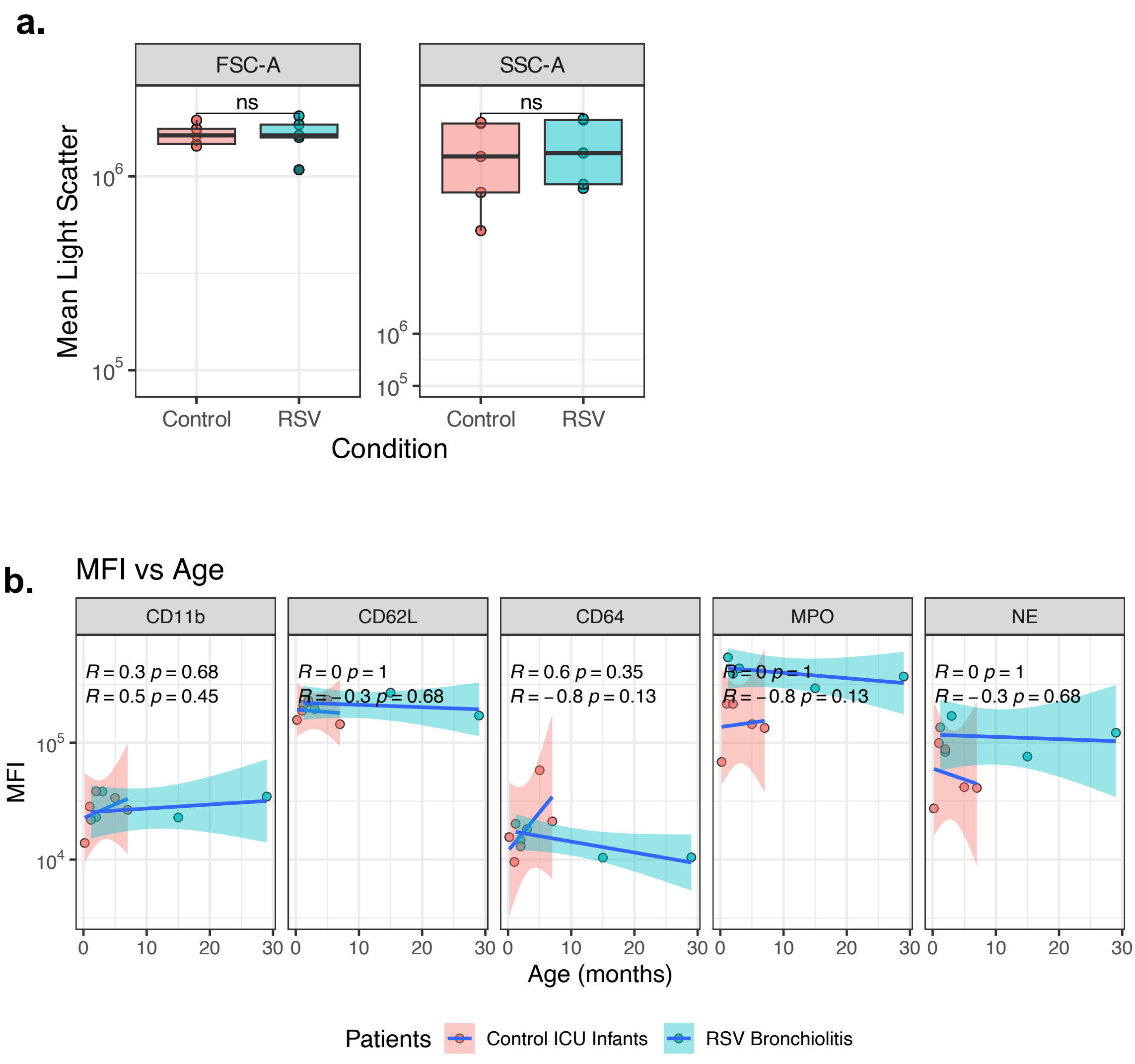

Supplementary Figure 4. Additional Analyses of Infant Sample Variability. (a) Comparison of forward scatter (FSC-A) and side scatter (SSC-A) between infant control and infant RSV bronchiolitis samples. Boxplots display median and interquartile range (n=5 biologically independent samples) ns = non-significance using unpaired t test. (b) Correlation plots showing the relationship between infant age and mean fluorescence intensity (MFI) of neutrophil activation markers, showing no age-associated trends within infant samples. (n=5 biologically independent samples per group).

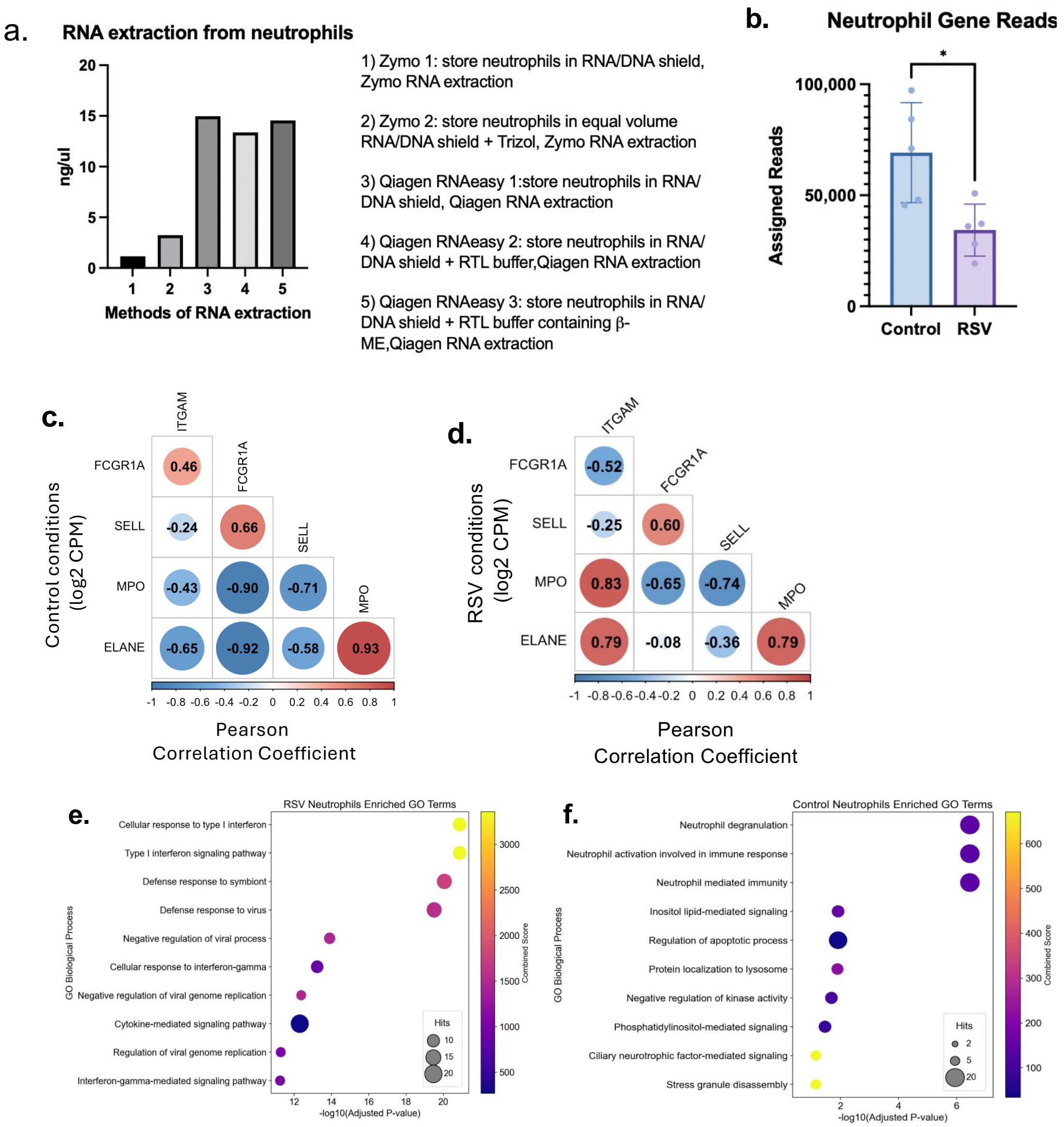

Supplementary Figure 5. Transcriptomics Quality Control. (a) Comparison of five different neutrophil RNA extraction methods (n=1 technical replicate per method). (b) Number of assigned sequencing reads in purified neutrophils from control and RSV-infected infants. Bars show mean ± SD, demonstrating reduced transcript capture in RSV samples. (n=5 biologically independent samples). Statistical significance was assessed using Mann-Whitney U test (control vs RSV), $p < 0.05$. (c) Pearson correlation matrix of key neutrophil genes under control conditions ($\log_2$ CPM). Higher positive correlations are shown in red, negative correlations in blue. (d) Pearson correlation matrix of the same genes under RSV-infected conditions ($\log_2$ CPM). (e) Gene ontology (GO) enrichment analysis of pathways upregulated in RSV neutrophils. Dot plot displays the top enriched GO biological processes, with dot size indicating number of genes per pathway and colour indicating $-\log_{10}$ adjusted p-value. (f) GO enrichment analysis of pathways associated with control neutrophils.

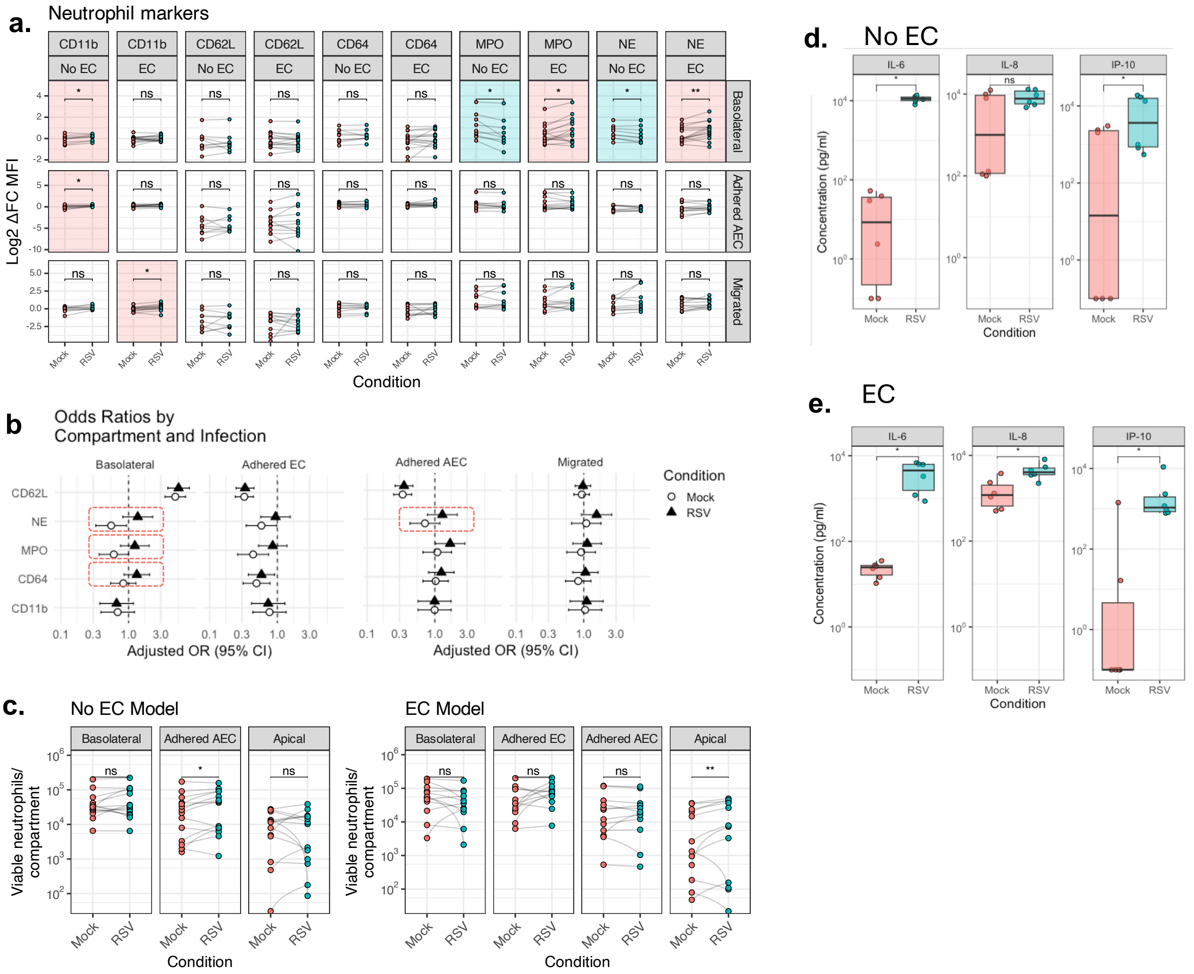

Supplementary Figure 6. Impact of Neutrophil Migration on Viral Load, Cytokine Production, and Neutrophil Distribution in Models with and without Endothelial Cells (EC). (a–b) Additional data visualisation outputs showing differences in infection, compartment or immune response. (n=6 No EC, n=9 EC independent experiments). (c) Distribution of neutrophils across model compartments (basolateral, adhered AEC, apical) in EC and No-EC systems under RSV and mock conditions; no significant differences observed. Mean ± SEM (n=6-9 independent experiments); (d) Viral load and cytokine concentrations (IL-6, IL-8, IP-10) in “No EC” epithelial-only models. Boxplots display median and interquartile range (n=6 independent experiments).(e) Viral load and cytokines in models incorporating endothelial cells. RSV significantly increases viral replication ($p < 0.01$) and cytokine production ($p < 0.05$) relative to mock. Boxplots display median and interquartile range (n=6 independent experiments). Statistical significance was assessed using a paired t-test (mock vs RSV); ns = not significant; *$p < 0.05$, **$p < 0.01$.

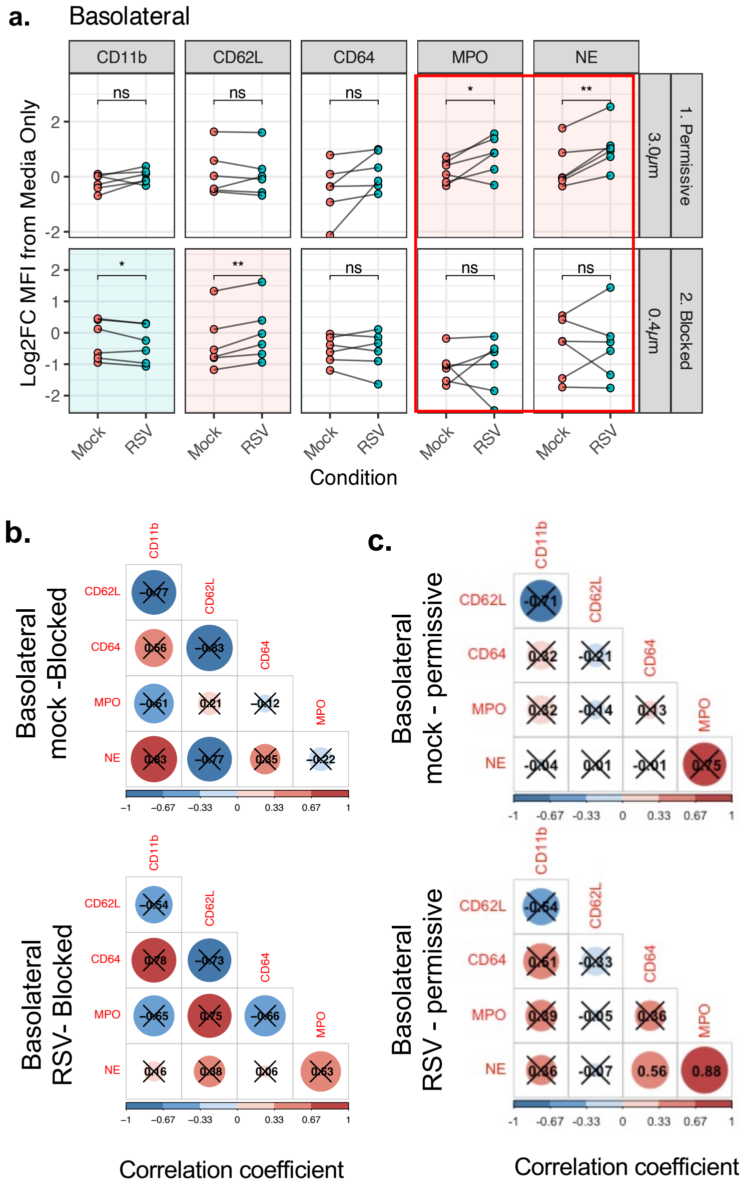

Supplementary Figure 7. Correlation of Neutrophil Marker Expression in the Basolateral Blocked Condition. (a) Paired analysis of neutrophil marker expression across experimental conditions, illustrating comparative changes in activation marker levels. (n=6 independent biological neutrophil samples) Statistical significance was assessed using a paired t-test (mock vs RSV); ns = not significant; *p < 0.05, **p < 0.01.; (b–c) Correlation matrices for neutrophil activation markers (CD11b, CD62L, CD64, MPO, NE) across different experimental conditions (mock-blocked, mock-permissive, RSV-permissive, RSV-blocked). Positive correlations are shown in red and negative in blue; only statistically significant correlations are displayed. Heatmaps illustrate how marker relationships vary depending on epithelial/endothelial permissiveness and infection status.

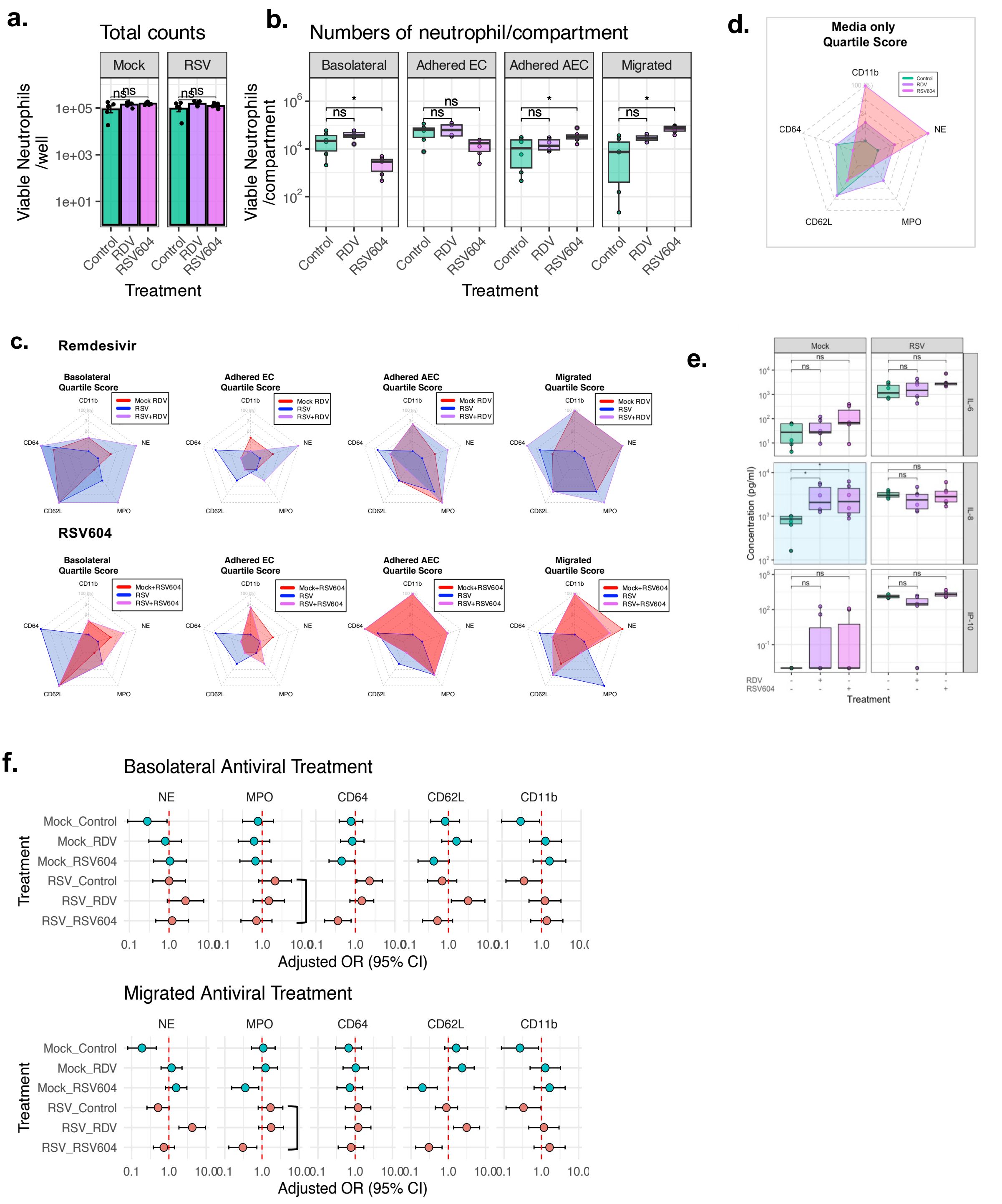

Supplementary Figure 8. Impact of Antiviral Treatments on Cytokine Production and Neutrophil Activation in RSV Models. (a) Total counts of neutrophils recovered from all compartments, demonstrating no loss of cell viability across treatment conditions. Bars show mean ± SEM (n=6 independent biological neutrophil samples); Statistical analysis was performed using paired t test between control:RDV or control: RSV604 conditions. (b) Total neutrophil counts separated by compartment (basolateral, adhered, migrated), confirming comparable recovery in each compartment. Boxplots display median and interquartile range (n=6 biologically independent neutrophil samples). Statistical analysis was performed using paired

wilcoxon test between control:RDV or control: RSV604 conditions. (c–d) Quartile-scaled radar plots of mean fluorescence intensity (MFI) for neutrophil activation markers (CD11b, CD62L, CD64, MPO, NE) in basolateral and migrated compartments under RSV604, Remdesivir (RDV), and control conditions. (e) Cytokine concentrations (IL-6, IL-8, IP-10) measured across Mock, RSV, RDV, and RSV604 treatment groups. Boxplots display median and interquartile range (n=6 biologically independent neutrophil samples). Statistical analysis was performed using paired wilcoxon test between control:RDV or control: RSV604 conditions. (f) Odds ratio plot showing relative likelihood of marker expression changes in RSV versus treatment with antivirals; points represent odds ratios with 95% confidence intervals (n=6 biologically independent samples per group); ns = not significant; *$p < 0.05$, **$p < 0.01$.

Supplementary Table 1 The oligonucleotide sequences used in this study.

Primer/Probe	Sequence 5’-3’
**RSV-A Forward Primer**	CTCAATTTCCTCACTTCTCCAGTGT
**RSV-A Reverse Primer**	CTTGATTCCTCGGTGTACCTCTGT
**RSV-A Probe**	[6FAM] TCCCATTATGCCTAGGCCAGCAGCA [TAMRA]

Supplementary Table 2 Dilutions and catalogue numbers for all antibodies used.

Antibody	Target	Fluorophore	Supplier	Catalog No.	Dilution
**CD11b**	Integrin	FITC	Miltenyi Biotec	130-110-552	1/50
**CD62L**	L-selectin	PE-Cy7	Miltenyi Biotec	130-129-810	1/50
**CD64**	Fc receptor	APC-Cy7	Miltenyi Biotec	130-116-199	1/50
**MPO**	Secreted effector enzyme	APC	Miltenyi Biotec	130-119-786	1/50
**NE**	Secreted effector enzyme	PE	Santa Cruz Biotechnology	sc-55549 PE	1/50
