# Supplementary material for: Neutrophil myeloperoxidase as a functional biomarker for RSV severity: implications for in vitro therapeutic screening
Source: Nat Commun. 2026 Jul 13;17:5507. doi: 10.1038/s41467-026-74414-0 (PMC13365213; doi:10.1038/s41467-026-74414-0)
Supplement: Supplementary file 2 — Description of Additional Supplementary Files [file 41467_2026_74414_MOESM2_ESM.pdf]

Description of Additional Supplementary Files

Supplementary Video 1 Timelapse video of neutrophils (red) interacting with mock-infected epithelial cultures. Imaged every 2 minutes for 1 hour from the addition of neutrophils using an inverted Zeiss LSM 710 confocal microscope. Scale bars, as indicated.

Supplementary Video 2 Timelapse video of neutrophils (red) interacting with RSV-infected epithelial cultures. RSV-infected epithelial cells are shown in green. Imaged every 2 minutes for 1 hour from the addition of neutrophils using an inverted Zeiss LSM 710 confocal microscope. Scale bars, as indicated.

Supplementary Video 3 High-speed video (supplementary video 3) demonstrating ciliary motility and epithelial function of ALI cultures.
