# Supplementary material for: Neutrophil myeloperoxidase as a functional biomarker for RSV severity: implications for in vitro therapeutic screening
Source: Nat Commun. 2026 Jul 13;17:5507. doi: 10.1038/s41467-026-74414-0 (PMC13365213; doi:10.1038/s41467-026-74414-0)
Supplement: Supplementary file 6 — Reporting Summary [file 41467_2026_74414_MOESM6_ESM.pdf]

nature portfolio

Corresponding author(s): Claire M Smith

Last updated by author(s): Apr 21, 2026

Reporting Summary

Nature Portfolio wishes to improve the reproducibility of the work that we publish. This form provides structure for consistency and transparency in reporting. For further information on Nature Portfolio policies, see our Editorial Policies and the Editorial Policy Checklist.

Statistics

For all statistical analyses, confirm that the following items are present in the figure legend, table legend, main text, or Methods section.

n/a	Confirmed	
☐	☒	The exact sample size (*n*) for each experimental group/condition, given as a discrete number and unit of measurement
☐	☒	A statement on whether measurements were taken from distinct samples or whether the same sample was measured repeatedly
☐	☒	The statistical test(s) used AND whether they are one- or two-sided<br>*Only common tests should be described solely by name; describe more complex techniques in the Methods section.*
☐	☒	A description of all covariates tested
☐	☒	A description of any assumptions or corrections, such as tests of normality and adjustment for multiple comparisons
☐	☒	A full description of the statistical parameters including central tendency (e.g. means) or other basic estimates (e.g. regression coefficient) AND variation (e.g. standard deviation) or associated estimates of uncertainty (e.g. confidence intervals)
☐	☒	For null hypothesis testing, the test statistic (e.g. *F*, *t*, *r*) with confidence intervals, effect sizes, degrees of freedom and *P* value noted<br>*Give P values as exact values whenever suitable.*
☒	☐	For Bayesian analysis, information on the choice of priors and Markov chain Monte Carlo settings
☒	☐	For hierarchical and complex designs, identification of the appropriate level for tests and full reporting of outcomes
☐	☒	Estimates of effect sizes (e.g. Cohen's *d*, Pearson's *r*), indicating how they were calculated

Our web collection on statistics for biologists contains articles on many of the points above.

Software and code

Policy information about availability of computer code

Data collection	No specific code was used in the data collection
Data analysis	Flow cytometry analysis was performed using FlowJo v10<br>Data manipulation and visualization was carried out using Python 3.12.2 and R 4.4.0<br>Statistical analysis was perfomed using R 4.4.0 and GraphPad Prism v11<br><br>Custom code for the analysis performed in this study is publicly available via GitHub at https://github.com/smithlab-code/NCOMMS-25-82134.git

For manuscripts utilizing custom algorithms or software that are central to the research but not yet described in published literature, software must be made available to editors and reviewers. We strongly encourage code deposition in a community repository (e.g. GitHub). See the Nature Portfolio guidelines for submitting code & software for further information.

Data

Policy information about availability of data

All manuscripts must include a data availability statement. This statement should provide the following information, where applicable:

- Accession codes, unique identifiers, or web links for publicly available datasets
- A description of any restrictions on data availability
- For clinical datasets or third party data, please ensure that the statement adheres to our policy

RNA-seq data generated in this study have been deposited in the European Genome-Phenome Archive (EGA) under accession code XXXXX(will be emailed to editor) (https://ega-archive.org/). Processed RNA-seq data and all other data supporting the conclusions of this study are provided within the Article, Supplementary Information, or the accompanying Source Data file. Source data are provided with this paper.

Research involving human participants, their data, or biological material

Policy information about studies with human participants or human data. See also policy information about sex, gender (identity/presentation), and sexual orientation and race, ethnicity and racism.

Reporting on sex and gender	We ensured all primary human samples were age and sex matched. HUVECs were from mixed donors. HEp-2 (Human Epithelial type 2) cells purchased from ATCC (Cat# CCL23) were female.
Reporting on race, ethnicity, or other socially relevant groupings	Population characteristics are described in Table 1 and 2
Population characteristics	Population characteristics are described in Table 1 and 2
Recruitment	Participants were recruited from Great Ormond Street Hospital NHS Foundation Trust from March 2022 to February 2024. Exclusion criteria for the cohort included current smokers, active haematological malignancies or cancer, known immunodeficiencies, sepsis from any cause and blood transfusions within 4 weeks, known bronchial asthma, diabetes, hay fever, and other known chronic respiratory diseases such as cystic fibrosis, interstitial lung disease and chronic obstructive pulmonary disease. Nasal brushings and blood were obtained by trained clinicians from healthy children and adults that reported no respiratory symptoms in the preceding 7 weeks. All methods were performed in accordance with the relevant guidelines and regulations.
Ethics oversight	Ethical approval for use of paediatric blood samples and paediatric AECs was given through the Living Airway Biobank, administered through the UCL Great Ormond Street Institute of Child Health (REC reference: 19/NW/0171, IRAS project ID: 261511, Northwest Liverpool East Research Ethics Committee). Ethical approval for the collection and use of venous blood from healthy adult volunteers was obtained from the UCL Research Ethics Committee (Project ID: 19165/001)

Note that full information on the approval of the study protocol must also be provided in the manuscript.

Field-specific reporting

Please select the one below that is the best fit for your research. If you are not sure, read the appropriate sections before making your selection.

[x] Life sciences [ ] Behavioural & social sciences [ ] Ecological, evolutionary & environmental sciences

For a reference copy of the document with all sections, see nature.com/documents/nr-reporting-summary-flat.pdf

Life sciences study design

All studies must disclose on these points even when the disclosure is negative.

Sample size	Samples sizes were calculated based on our previous studies using ALI cultures grown from donor material from different disease groups. Using a conservative estimated minimum effect size of 40% for a difference in viral titres between drug conditions with an estimated standard deviation of 30%, a desired power of 80% and a p-value of < 0.05 for statistical significance, a sample size is 5 donors per group per experiment was needed.
Data exclusions	ID11 was of insufficient quality and was excluded from the flow cytometry analysis
Replication	All experiments were repeated with at least 2 technical repeats. Experiments on human neutrophils were repeated from at least 5 independent donors. Human airway epithelial cells were repeated from at least 6 independent donors.
Randomization	Randomization was not applicable in this study because comparisons were performed on paired samples from the same individual, with each participant serving as their own control. Samples were processed and analysed using identical, predefined protocols, and no allocation to independent experimental groups was undertaken.
Blinding	Blinding was not applied because outcomes were measured using objective, automated flow cytometry readouts with predefined gating strategies, and therefore did not involve subjective assessment. In addition, statistical analyses were performed on paired samples from the same individual, further reducing the potential for bias related to sample identity.

Reporting for specific materials, systems and methods

We require information from authors about some types of materials, experimental systems and methods used in many studies. Here, indicate whether each material, system or method listed is relevant to your study. If you are not sure if a list item applies to your research, read the appropriate section before selecting a response.

Materials & experimental systems

n/a	Involved in the study
☐	☒ Antibodies
☐	☒ Eukaryotic cell lines
☒	☐ Palaeontology and archaeology
☒	☐ Animals and other organisms
☒	☐ Clinical data
☒	☐ Dual use research of concern
☒	☐ Plants

Methods

n/a	Involved in the study
☒	☐ ChIP-seq
☐	☒ Flow cytometry
☒	☐ MRI-based neuroimaging

Antibodies

Antibodies used

All antibodies used in this study were commercially sourced and used at a dilution of 1:50. Antibodies used include anti-CD11b–FITC (Miltenyi Biotec, catalog no. 130-110-552), anti-CD62L–PE-Cy7 (Miltenyi Biotec, catalog no. 130-129-810), anti-CD64–APC-Cy7 (Miltenyi Biotec, catalog no. 130-116-199), anti-myeloperoxidase (MPO)–APC (Miltenyi Biotec, catalog no. 130-119-786), and anti-neutrophil elastase (NE)–PE (Santa Cruz Biotechnology, catalog no. sc-55549 PE). Antibodies were used according to the manufacturers' recommendations.

Validation

All antibodies used were commercial antibodies.

Eukaryotic cell lines

Policy information about cell lines and Sex and Gender in Research

Cell line source(s)

Murine embryonic 3T3-J2 fibroblasts originally sourced from Simon Broad, Prof. Fiona Watt (Kings College London, London, UK), Dr. Paola Bonfanti (University College London, London, UK), and Prof. Howard Green (Harvard Medical School, Boston, MA).

HUVEC transduced with ETS variant transcription factor 2, also known as 'reset' vascular ECs prodvided by P.De Coppi as decribed Palikuqi B, Nguyen DT, Li G, Schreiner R, Pellegata AF, Liu Y, et al. Adaptable haemodynamic endothelial cells for organogenesis and tumorigenesis. Nature. 2020;585(7825):426-32.

HEp-2 (Human Epithelial type 2) cells were purchased from ATCC (Cat# CCL23)

Authentication

None of these cell lines were authenticated.

Mycoplasma contamination

All cell lines and primary cells were confirmed negative for mycoplasma contamination by PCR testing

Commonly misidentified lines (See ICLAC register)

none

Plants

Seed stocks

not used

Novel plant genotypes

not used

Authentication

not relevant

Flow Cytometry

Plots

Confirm that:

- [x] The axis labels state the marker and fluorochrome used (e.g. CD4-FITC).
- [x] The axis scales are clearly visible. Include numbers along axes only for bottom left plot of group (a 'group' is an analysis of identical markers).
- [x] All plots are contour plots with outliers or pseudocolor plots.
- [x] A numerical value for number of cells or percentage (with statistics) is provided.

Methodology

Sample preparation	Neutrophils were centrifuged, blocked with Human TruStain FcX™, and stained with LIVE/DEAD™ fixable violet dye (Thermo Fisher Scientific, UK). Following washing, cells were stained with 1/50 dilution of CD11b-FITC (Miltenyi Biotec, 130-110-552), CD64-APC-Cy7 (Miltenyi Biotec, 130-116-199), and CD62L-PE-Cy7 (Miltenyi Biotec, 130-129-810) for 20 minutes at 4°C in the dark. Cells were washed, fixed with PFA, permeabilised, and stained intracellularly with MPO-APC (Miltenyi Biotec, 130-119-786) and NE-PE (Santa Cruz Biotechnology, sc-55549 PE). After a final wash, neutrophils were resuspended in FACS buffer
Instrument	CytoFLEX S, Beckman Coulter, USA
Software	FlowJo v10
Cell population abundance	100,000 - 500,000
Gating strategy	The main cell population was first gated based on FSC-A and SSC-A to exclude debris. From these events, single cells are isolated using FSC-A versus FSC-H to remove doublets. A live-cell gate is then applied using the viability-dye channel to exclude dead cells. Finally, CD11b expression is assessed within the live-singlet population, using the unstained control to define the negative boundary and the stained sample to identify the CD11b-positive subset. Same-day “medium-only” controls and use FMO and cytometer calibration/PMT stability checks.

- [x] Tick this box to confirm that a figure exemplifying the gating strategy is provided in the Supplementary Information.
