# Supplementary material for: Neutrophil myeloperoxidase as a functional biomarker for RSV severity: implications for in vitro therapeutic screening
Source: Nat Commun. 2026 Jul 13;17:5507. doi: 10.1038/s41467-026-74414-0 (PMC13365213; doi:10.1038/s41467-026-74414-0)
Supplement: Supplementary file 7 — Transparent Peer Review File [file 41467_2026_74414_MOESM7_ESM.pdf]

nature portfolio

Peer Review File

Neutrophil myeloperoxidase as a functional biomarker for RSV severity: implications for in vitro therapeutic screening.

Corresponding Author: Dr Claire Smith

This file contains all reviewer reports in order by version, followed by all author rebuttals in order by version.

Version 0:

Reviewer comments:

Reviewer #1

(Remarks to the Author)
Neutrophils play an important pathological role in RSV disease severity, possibly in relation to “pus” and mucous plugging. The authors therefore studied neutrophil from infants with severe RSV bronchiolitis admitted to the PICU, and found elevated activation markers (MPO, NE). They then built a pediatric airway epithelial–endothelial co-culture model and added human neutrophils to study how RSV infection triggers neutrophil activation and migration. Using this system, they tested two antivirals (Remdesivir, or RDV and RSV604) and showed that both reduced viral replication, but only RSV604 dampened neutrophil degranulation (MPO/NE). They propose that MPO is a functional biomarker of neutrophil activation in RSV infection. The model is very elegant, and relevant to clinical RSV diseases.

The manuscript is well written and the experiments are detailed. The use of peripheral blood samples from infants with RSV is important to support in vivo relevance. Nonetheless, I have a couple of major concerns:

1) A fundamental premise of the article is the increase levels of MPO and NE in RSV-infected compared to controls. However, first the numbers are small (N=5 per group), the controls are children with complex conditions, and most importantly, the RSV-infected infants are much older, and we know from the literature that both MPO and NE are lower in younger, compared to older infants. They acknowledge this as a limitation, but this is a fundamental issue. The conclusion that MPO is a biomarker of RSV severity is not supported. To be convinced, I would like to see that levels of MPO and NE remain higher in RSV-infected infants after adjusting for age. I would also like to see that the difference still holds in a younger group of RSV-infected infants more comparable to the controls.

2) AECs are generated from a single 2-year-old donor, and the neutrophils come from adults. Can the author provide evidence that at least the key results (e.g. effect of ECs; neutrophil trans-epithelial migration increasing following RSV infection suggesting a key role in RSV pathogenesis) are replicated when using neonatal neutrophils (e.g. from cord blood)? Also, whether neutrophil experiments are technical or biological replicates is unclear. Given the known heterogeneity of neutrophils responses in children, the data should reflect multiple (at least 3, ideally 4-6?) neutrophil donors in each experiment.

3) The study reports differences with two antiviral drugs on neutrophil degranulation markers. They emphasize the reduced neutrophil MPO with RSV604. This drug has been studied in clinical trials but has never been pursued commercially. RDV does inhibit viral load in vivo, but in their model, it did not affect MPO (in fact Figure 5f shows that RDV may increase MPO activity). Without more mechanistic experiments, and evidence that neonatal neutrophils behave similarly (at least for the key results) what is the importance and significance of the drug-testing findings?

4) Can the authors expand on the molecular mechanism by which direct contact with RSV-infected epithelial cells leads to neutrophil activation. While the authors show that physical proximity is required, the specific epithelial signals or receptor–ligand interactions involved (e.g., adhesion molecules, danger signals, surface-bound viral antigens) are not discussed. To gain more mechanistic insights, the authors may want to consider blocking neutrophil / AECs interactions by inhibiting surface molecules using specific antibodies, or gain/loss-of-function experiments, to see if they reverse the neutrophil activation phenotype.

5) The findings in the reverse migration section are based on migration inhibition using 0.4um diameters membrane pores

instead of 3um. Reverse migration has well-defined molecular markers (none measured here). See PMC9686117 for example. What they are measuring may not be related to reverse migration. However, findings that reverse migration affects CD62L, MPO and NE expression is relevant in the context of RSV infection. Could the author link these in vitro findings to the pediatrics neutrophils phenotype in vivo? They could consider examining the signature of neutrophils in their in vitro ALI system, using RNA sequencing, and compare this signature to published neutrophil datasets from RSV infected vs control infants.

Minor comments:
1) The abstract should present and integrate the key results.
2) The authors use many abbreviations that makes the article harder to read. Please make sure that each abbreviation is at least expanded on first use.
3) Can the author explain how they justify the doses of RSV604 or RDV used?
4) Claims about antiviral potency based on non-infectious viral RNA levels can be misleading since they are measuring viral load, not infection (and their infection assay lacks sensitivity). The significance of the findings in Figure 4 needs further explanation.
5) Page "Meanwhile, RSV infection resulted in a significant >5-fold increase in IL-6, and IP-10 secretion relative to untreated controls (Fig. 4e and Supplementary Figure 5)." – Are the authors referring to the correct figure panels? Should it not be Fig. 4f-g?

(Remarks on code availability)

Reviewer #2

(Remarks to the Author)
The manuscript by Palor et al. investigates neutrophil activation and degranulation in the context of RSV infection, focusing on myeloperoxidase (MPO) levels as a functional readout of therapeutic efficacy. The study is based on the observation that RSV infection promotes neutrophil recruitment and activation in the lungs and increases activation markers on circulating neutrophils. To address this, the authors analyzed neutrophils in whole blood from RSV-infected children and assessed neutrophil functions and phenotypes using a human trans-epithelial air–liquid interface model incorporating endothelial cells. Additionally, they examined the effects of two antiviral drugs, remdesivir and RSV604, on neutrophil migration and activation. Their experiments showed that while both compounds reduced viral load, only RSV604 attenuated MPO expression. Based on these findings, the authors propose that MPO may serve as a potential indicator of disease severity in RSV infection and as a functional marker of therapeutic efficacy.
The manuscript is generally well written; however, I have several concerns that require clarification, and further mechanistic studies are needed to substantiate the study's conclusions.

Comments
1: Fig1: The authors analyzed peripheral blood neutrophils from RSV-infected infants and found that circulating neutrophil counts were lower in these patients. Interestingly, activation markers such as CD11b, CD62L, and CD64 were not upregulated, yet expression levels of MPO and NE in these neutrophils were increased. How do the authors explain this apparent discrepancy? Previous studies, as noted in the introduction, have reported increased expression of these activation markers on neutrophils from RSV-infected infants (ref. 12). Is the observed increase in MPO due to de novo expression—i.e., are MPO mRNA levels also elevated? Furthermore, how does this relate to plasma MPO and/or NE concentrations in these infants?

2: Fig 2: The addition of an endothelial cell layer to the AEC-ALI is interesting and innovative. However, the authors seem to draw conclusions rather quickly from these experiments. For example:
"Initial experiments indicated that incorporating ECs into AEC cultures (see timeline in Fig. 2a) maintained TEER values above 200 $\Omega \cdot cm^2$ (Supplementary Fig. 1a) and markedly reduced dextran permeability across the membrane insert (Supplementary Fig. 1b). Based on this, they suggest that ECs improve the tightness and selective permeability of the epithelial barrier."
However, I believe the effect could also be more straightforward; the endothelial layer likely serves as an extra physical barrier, which limits FITC-dextran movement toward the apical side.
A similar reasoning applies to Figure 2h: in my view, endothelial cells likely primarily act as a barrier, preventing migration into the apical region.
The experiments (Figures 2b–f) indicate that in AEC models containing endothelial cells, pro-inflammatory cytokine levels are lower before neutrophil migration but become higher after migration compared to models without endothelial cells. It remains unclear whether these changes result from cytokine consumption or increased cell activation. Moreover, since endothelial cells themselves can produce these cytokines, it would be of interest to investigate their contribution.
In Figure 2J, the authors report that MPO and NE appear "upregulated" in neutrophils following RSV infection in AEC models containing endothelial cells. However, the term "upregulated" is potentially confusing, as it seems unlikely that neutrophils synthesize additional MPO or NE. A more plausible explanation could be that these proteins, which are stored in primary granules, become more accessible for detection by flow cytometry upon neutrophil activation or priming under these conditions. If the authors claim that neutrophils actually contain higher levels of MPO, this should be validated using a quantitative assay such as ELISA.

3: Figure 3:
In Figure 3, the authors present experiments using either permissive or blocking inserts to assess the impact of neutrophil

migration on MPO and NE levels in the basolateral compartment. The data suggest that basolateral neutrophils display higher MPO and NE levelsin RSV-infected cultures under permissive conditions compared to blocked conditions. The authors interpret these findings as evidence for reverse migration of neutrophils. However, this conclusion seems premature given the current system and data. There is no direct evidence of true reverse transmigration, and the observed differences could also result from neutrophil adhesion. Furthermore, similar to Figure 2, the analysis is limited to a single time point (1 hour after neutrophil addition). Time-course experiments would provide more robust insights into the dynamics of neutrophil adhesion and migration linked to phenotype and function.
In Figure 3h, the authors present results from enzyme activity assays (these are not ELISAs as the authors state) on basolateral supernatants, showing increased MPO activity—but not NE activity—following RSV infection under permissive conditions. For NE, this appears inconsistent with the data in Figure 3f; however, the discrepancy could reflect differences in assay sensitivity and the relative abundance of MPO versus NE in neutrophils. Measuring protein content with an ELISA would be preferred.

4: In Figure 4, a series of experiments is shown that assess the impact of antiviral treatment on viral load, cytokine production, and neutrophil marker expression in AEC models that include endothelial cells. The section title suggests that these treatments reduce viral load while increasing baseline inflammatory cytokine production. While the reduction in viral load is evident, the meaning of “enhanced baseline cytokine production” is unclear, as there appears to be no substantial difference in cytokine levels between the RSV conditions. It is also not specified whether these experiments were conducted under permissive or blocked conditions. Furthermore, although the introduction states that the effects of the drugs on neutrophil marker expression were examined, this is not apparent from Figure 4. Finally, were the drugs tested for epithelial and endothelial cell and, neutrophil toxicity?

5: In Figure 5, data are shown illustrating the effects of antiviral drugs on neutrophil degranulation markers (MPO and NE). RSV604 markedly reduced nearly all degranulation and activation markers, whereas RDV showed only modest effects. Additional, mostly modest, changes were observed in neutrophil viability, MPO activity, and cytokine levels. Interpretation of these findings is challenging due to the small sample size and high variability among data points. Moreover, the underlying mechanism remains unclear: do these drugs act directly on neutrophils, or are the effects mediated indirectly through endothelial or epithelial cells? Examining the direct effects of the drugs on these cells could provide more insights into the underlying mechanisms.

Additional comments:

- Abstract is confusing and needs to be rewritten. It refers to ‘these clinical investigations’ but it is not clear what ‘these’ are. In addition, the research objective mentioned in the abstract (focused on MPO) does not match the aim formulated in the introduction (focused on neutrophil activation and degranulation). Also, what is meant by “MPO-driven neutrophil activation”? This suggests an active role for MPO in neutrophil activation but was not studied here.

- The time between drawing blood and the isolation of neutrophils can have a major impact on the analysis of neutrophil function and activation but is not mentioned. This should be clarified in the methods sections. Was this standardized between patients and controls?

- The expression of MPO and neutrophil activation markers is reported as mean fluorescence intensity (MFI), which can be challenging to compare across different measurement days due to variability in flow cytometer settings. Reliable comparison requires normalization using calibration beads or reference samples; however, I did not find any description of such procedures in the methods section.

- I find the description of the trans-epithelial migration assay in the methods section somewhat unclear, which makes it difficult to assess exactly how they analysed migration. Please elaborate on the method used.

- “Purified neutrophils were diluted to 5 x 106 cells/ml in HBSS+/+ with 1% (v/v) autologous human serum. 100 ul was added basolaterally to each membrane insert and incubated for one hour”
Is there a specific reason for choosing this number of neutrophils and timeframe? Does this have some physiological basis? Have these conditions been optimized in pilot experiments?

- The radarplot in figure 1i is redundant as figure 1c already clearly shows the differences between control ICU and RSV infected infants.

- In the discussion, the authors acknowledge as a limitation of their in vitro studies the use of adult neutrophils, noting that only minor differences in resting state and subtle variations in neutrophil responsiveness might occur. However, in a recent paper comparing infant and adult neutrophils (ref. 11), the same authors appear to take a different view, stating that these studies suggest, “….. intrinsic differences in neutrophil responses between adults and infants, which may partly explain the severe bronchiolitis observed in infants”. Please clarify.

(Remarks on code availability)

Reviewer #3

(Remarks to the Author)
I co-reviewed this manuscript with one of the reviewers who provided the listed reports. This is part of the Nature Communications initiative to facilitate training in peer review and to provide appropriate recognition for Early Career Researchers who co-review manuscripts.

(Remarks on code availability)

Version 1:

Reviewer comments:

Reviewer #1

(Remarks to the Author)
The authors have addressed well my comments and have considerably improved the article. The system is very elegant, and the results are exciting and extensive.

However, the following concerns remain:

1) Whether MPO/NE is a biomarker of severe RSV disease or not.

The authors present additional data in Supplemental Figure 4b, showing the MPO and NE remain elevated in children with severe RSV even at a young age. However, their conclusion that “These findings establish MPO as a biomarker of RSV disease severity” is overstated. First, this study was not designed to establish the usefulness of this biomarker. Second, the main MPO observation in children with severe disease stands on few in each group tested at unspecified times during their PICU stay, who also have heterogenous clinical conditions. Therapeutic efficacy was established in a model that uses older pediatric airway cells and adults rather than cord blood neutrophils.

This is misleading. I recommend changing the abstract to (with specific suggestions underlined):

“Respiratory syncytial virus (RSV) is a leading cause of severe lower respiratory tract infections in infants, yet therapeutics are lacking. The aim of this study is to develop an in vitro model that recapitulates key clinical outcomes in infants with RSV bronchiolitis to help the discovery of effective therapeutics. Neutrophil activation and influx into the airways are hallmarks of severe RSV infection, but these responses are difficult to quantify in clinical trials. Here we profile peripheral blood neutrophils from infants with RSV admitted to the Paediatric Intensive Care Unit (PICU). MPO was elevated in a clinically heterogenous group of children with severe RSV disease, compared to age-matched controls. To mechanistically model this response, we established a paediatric airway epithelial air–liquid interface (ALI) system incorporating an endothelial layer and primary neutrophils from adults, to recapitulate the tissue microenvironment at the blood-airway barrier. Following RSV infection, neutrophil migration and activation were assessed using flow cytometry. The inclusion of an endothelial layer enhanced physiological relevance and more accurately replicated in vivo MPO responses. We then evaluated two antiviral candidates (remdesivir (RDV) and RSV604) to assess their ability to modulate neutrophil activation. While both compounds reduced viral load at 24 hours post-infection, only RSV604 attenuated MPO expression. This model suggests that MPO could be useful as a readout of therapeutic efficacy. Targeting neutrophil-driven inflammatory pathways may be critical for reducing pathology in infant RSV infection.”

2) Data were generated using a single 2-year-old pediatric airway donor.

This is another major limitation. Given the expected variability in humans, it is unfortunate that the results were reproduced technically but not biologically using only one pediatric cell source. While the system is very elegant this was not sufficiently acknowledged in the limitations. First, the figures should clearly indicate the number of technical versus biological replicates, including the cell source and N= for each experimental replicate. Second, the limitation section should acknowledge that using this system for therapeutic drug screening in the future should use pediatric cells (or at least confirm whether findings from this study should be validated using pediatric neutrophils).

(Remarks on code availability)
I have not reviewed the code.

Reviewer #2

(Remarks to the Author)
The authors have responded thoroughly to my comments, provided additional data to support their conclusions, and tempered some of their earlier overinterpretations. This has improved the manuscript considerably.

(Remarks on code availability)

Reviewer #4

(Remarks to the Author)
I co-reviewed this manuscript with one of the reviewers who provided the listed reports. This is part of the Nature Communications initiative to facilitate training in peer review and to provide appropriate recognition for Early Career Researchers who co-review manuscripts.

(Remarks on code availability)

Open Access This Peer Review File is licensed under a Creative Commons Attribution 4.0 International License, which permits use, sharing, adaptation, distribution and reproduction in any medium or format, as long as you give appropriate credit to the original author(s) and the source, provide a link to the Creative Commons license, and indicate if changes were made.
In cases where reviewers are anonymous, credit should be given to 'Anonymous Referee' and the source.
The images or other third party material in this Peer Review File are included in the article’s Creative Commons license, unless indicated otherwise in a credit line to the material. If material is not included in the article’s Creative Commons license and your intended use is not permitted by statutory regulation or exceeds the permitted use, you will need to obtain permission directly from the copyright holder.
To view a copy of this license, visit https://creativecommons.org/licenses/by/4.0/

Response to Reviewers comments Nature Communications manuscript NCOMMS-25-82134

We thank the Reviewers for their thoughtful assessment and constructive suggestions. We have revised the manuscript substantially to address all points. Below, each comment is quoted and followed by our response. All textual changes are marked in the tracked-changes file.

We believe these comprehensive revisions strengthen the manuscript substantially. We thank the Reviewers for guidance that markedly improved the clarity, scope, and reproducibility of our work.

Reviewer #1

Major comments

1) “A fundamental premise is increased MPO/NE in RSV vs controls; numbers are small, controls are complex, and RSV infants are older. Please adjust for age and test whether differences persist; also test a younger RSV group comparable to controls.”

Response: We thank the reviewer for this insight, and we have now addressed age explicitly. We have re-analysed clinical flow cytometry with age adjustment (age in months) using generalized linear models with age in months as a covariate. MPO and NE remain significantly higher in RSV-infected infants after age adjustment (Results, Fig. 1f–g; Supplementary Fig. 4b; Methods-Statistics).

2) “AECs from a single 2-year-old donor and adult neutrophils. Replicate key results with neonatal neutrophils (e.g., cord blood) and ensure biological replication (≥3, ideally 4–6 donors).”

Response: We are grateful for this thoughtful suggestion, and we completely agree that incorporating infant or cord-blood neutrophils would be ideal from a biological standpoint. Unfortunately, routine inclusion of healthy neonatal neutrophils proved logistically impractical within the time scale required for the assays and obtaining fresh healthy infant neutrophils exactly 24 hours after RSV infection and drug treatment is not a feasible approach for a high throughput antiviral screening platform. With this limitation in mind, we focused on addressing the core biological concern raised by the reviewer- the influence of age. Because viability and activation state change rapidly ex vivo, neutrophils must be collected immediately before the migration assay. For ethical, logistical, and clinical-workflow reasons, this is not a sustainable or scalable approach for a screening platform intended for what we hope will become a high-throughput platform for antiviral testing.

With this limitation in mind, we focused on addressing the core biological concern raised by the reviewer - the influence of age. We directly compared adult and child neutrophils in Fig. 1f and found only difference in baseline CD64 expression, which was higher in control infants. These data show that adult neutrophils could act as a substitute in terms of CD11b, CD62L, MPO and NE. We have also introduced new experiments directly comparing paediatric and adult epithelial ALI cultures while

keeping the neutrophil donor population constant. These data show significantly greater neutrophil migration and selectively higher MPO signal in the paediatric epithelium condition (Results, Fig. 3h–j), highlighting the central importance of the paediatric airway microenvironment in dictating the neutrophil response. This finding also strengthens our rationale that, for screening purposes, paediatric epithelial context is the dominant variable, and adult neutrophils remain a practical and scientifically acceptable substitute.

We have expanded the Discussion and Limitations to state that future mechanistic or translational studies will prioritise the inclusion of neonatal neutrophils when feasible.

3) "Two antivirals: both reduce viral replication, only RSV604 reduces MPO. Without more mechanistic experiments and neonatal neutrophils, what is the importance/significance?"

Response: We thank the reviewer for raising this important point. We have softened our conclusions and now present the work clearly as a screening platform, where MPO provides an additional functional readout alongside viral load. The differing effects of RSV604 and RDV on MPO, despite similar reductions in viral replication, highlight how the model can distinguish candidates by their immunomodulatory profile, which is valuable for prioritising compounds before deeper mechanistic studies. To support careful interpretation, we also added time-course imaging and clarified that 24 h RT-qPCR quantifies viral genomes rather than infectious virus, with plaque assays at this time point below detection (Results, Fig. 6; and Methods).

4) "Expand on mechanism by which direct contact with RSV-infected epithelial cells leads to neutrophil activation; consider blocking Abs, GOF/LOF."

Response: We thank the reviewer for this suggestion, which we agree would add important mechanistic depth. While a full dissection of the epithelial-neutrophil signalling pathways is beyond the scope of this assay development-focussed study, we have taken steps to more directly address the reviewer's point. In the revised manuscript, we now demonstrate contact-dependence by comparing permissive (3 µm) and blocked (0.4 µm) inserts, showing that the increase in basolateral MPO/NE occurs only when neutrophils are permitted to interact directly with RSV-infected epithelium (Results, Fig. 5a–g). We have also added time-lapse imaging to illustrate neutrophil accumulation at infected epithelial sites (Results, Fig. 3d–f). Finally, we outline in the Discussion how blocking approaches (e.g., LFA-1/ICAM-1, selectins) could be incorporated in future mechanistic studies as we have done before (Herbert et al European Respiratory Journal 2020 56(2): 1902216; DOI: https://doi.org/10.1183/13993003.02216-2019).

5) "Reverse migration: conclusions are not supported; pore-size experiments may not reflect RM; consider linking in vitro to in vivo (e.g., RNA-seq comparison with published neutrophil datasets)."

Response: We thank the reviewer for this valuable observation. We have carefully softened our interpretation and now describe these findings as a basolateral phenotype consistent with cells that have contact with epithelial cells. We also clarify

that the pore-size experiment is intended to isolate migration-permissive contact, rather than to demonstrate reverse migration itself (Results, Fig. 5; Discussion). To strengthen the link to in-vivo biology, we have added neutrophil RNA-seq data from RSV-infected infants, which show enhanced degranulation-associated pathways (Results, Fig. 2f–j), and we discuss how these patterns align with our in-vitro MPO/NE findings.

Minor comments

1) “Abstract should present and integrate key results.”

Response: We have rewritten the Abstract to i) state the specific aim (develop an in vitro model that recapitulates key clinical outcomes), ii) summarize age-adjusted infant data (MPO/NE higher; activation markers unchanged), iii) highlight paediatric epithelium effects, and iv) clarify antiviral divergence on MPO (tracked changes in Abstract).

2) “Excessive abbreviations.”

Response: We expanded abbreviations on first use and shortened the list of abbreviations used (throughout manuscript)

3) “Justify doses of RSV604 or RDV.”

Response: We justify the concentrations used in the Methods section, citing prior literature for active ranges and noting that we also confirmed suppression of GFP-RSV spread by imaging through to day 7 pi (Results, Fig. 6d–e).

4) “Viral RNA measures may be misleading; infection assay lacks sensitivity; Figure 4 needs explanation.”

Response: We added explicit wording that RT-qPCR at 24 h quantifies viral genome copies, not necessarily infectious virus, and that plaque assays were below detection at that timepoint (Results, Fig. 6f text; Methods). We reframed interpretations accordingly.

5) “Figure panel references (IL-6/IP-10) appear incorrect (Fig. 4f–g?).”

Response: We have double checked all figure cross-references in Results and legends.

Reviewer #2

Major comments

1) “Fig 1: Circulating neutrophil counts lower; activation markers (CD11b, CD62L, CD64) not up; yet MPO/NE increased. Explain discrepancy. Is MPO de novo (mRNA)? Relation to plasma MPO/NE?”

Response: We thank the reviewer for this thoughtful point. We agree that increased intracellular MPO/NE can reflect granule mobilisation or priming, rather than de novo

synthesis, and we have therefore avoided the term "upregulated" throughout, instead referring to "higher MPO/NE signal by flow cytometry." We now clarify this explicitly in the Discussion.

To provide broader biological context, we have added neutrophil RNA-seq data from RSV-infected and control infants (Results, Fig. 2f–j), which show enrichment of degranulation-associated pathways, consistent with the flow-cytometry findings. We also reference prior reports of elevated plasma MPO/NE in RSV bronchiolitis as supportive background, while noting that we did not measure plasma proteins in this cohort and have highlighted this as a limitation and future direction.

2) "EC layer likely acts as a physical barrier; over-interpretation of tighter AEC barrier and reduced permeability; clarify cytokine sources; 'upregulated' MPO/NE is misleading-validate with ELISA."

Response: We appreciate this helpful clarification. In the revised text, we now emphasise that the endothelial layer primarily serves as an additional physical barrier/compartment, reducing dextran flux without altering RSV replication (Results, Fig. 4b–e). We have removed any implication of a tighter epithelial barrier. We also now present compartment-specific readouts and avoid attributing cytokine sources without cell-specific assays.

Regarding MPO/NE, we have replaced "upregulated" with more neutral terminology ("higher MPO/NE signal") and clarified the distinction between intracellular flow-cytometry measurements and extracellular enzymatic activity assays. We also corrected the prior mis-labelling and now accurately describe these as MPO/NE activity assays (Results, Fig. 4n; Methods).

3) "Figure 3-permissive vs blocking inserts: claims of reverse migration are premature; add time-course; reconcile MPO vs NE activity discrepancies; prefer ELISA for protein."

Response: We appreciate the reviewer's careful assessment. In response, we have softened the interpretation and now state that the higher basolateral MPO/NE signal observed under permissive pore conditions reflects a requirement for direct epithelial–neutrophil contact, rather than evidence of reverse migration. We have also added time-lapse imaging to illustrate the dynamics more clearly (Results, Fig. 3d–f).

Regarding MPO versus NE, we now note that differences in assay sensitivity and protein abundance likely account for the discrepancy, and we clarify this in the Results. Although we did not add ELISAs at this stage, we explicitly outline assay limitations and identify ELISA confirmation as an important future step (Discussion).

4) "Figure 4-'enhanced baseline cytokine production' unclear; specify permissive or blocked conditions; clarify whether drugs were tested for toxicity on epithelial/endothelial cells and neutrophils."

Response: We appreciate the reviewer's comments and have clarified this section accordingly. We now avoid the phrase "enhanced baseline" and instead present the cytokine data clearly by condition and compartment (Results, Fig. 4e, 4m), specifying permissive or blocked inserts wherever relevant.

To address the question of toxicity, we have added cell-viability assessments (LIVE/DEAD for neutrophils) and confirmed maintenance of ciliary activity and TEER in epithelial cultures under antiviral treatment (Methods; referenced in Results/legends). These data show no evidence of toxicity at the concentrations used over the 24-h period.

5) "Figure 5-drug effects on degranulation are modest/variable; mechanism unclear (direct vs indirect). Examine direct effects on each cell type."

Response: We appreciate the reviewer's thoughtful observation. In the revised text, we now present these findings as functional differences within the screening model, rather than mechanistic conclusions. We avoid implying any direct action of the antivirals on neutrophils and instead note that the changes in MPO/NE could reflect indirect effects via epithelial infection or signalling (Discussion). We have added a working model figure (Fig. 8) to illustrate this concept and outline targeted follow-up experiments.

Additional / Technical comments

"Abstract is confusing; mismatch of aims ('MPO') vs introduction ('neutrophil activation and degranulation'). 'MPO-driven activation' suggests causality."

Response: We have rewritten the Abstract to highlight study aims and removed causal phrasing such as "MPO-driven activation."

"Time from blood draw to neutrophil isolation not mentioned; standardize."

Response: Added to Methods-Neutrophil Isolation: all clinical samples were transferred immediately and processed upon arrival, with time-to-processing documented and minimized; adult donor samples were processed immediately after venipuncture.

"MFI comparability across days; need normalization/calibration."

Response: We added instrument/PMT stability checks, calibration bead runs, and analysis using paired fold-change to same-day medium-only controls to mitigate day variability (Methods-Flow cytometry; Statistics).

"Trans-epithelial migration assay unclear."

Response: We expanded the step-by-step migration assay description, including cell numbers (5×10^6 cells/mL; 100 µL basolateral addition; 1 h at 37 °C), insert orientation, collection of apical/adherent/basolateral fractions, and permissive vs blocked pore formats (Methods-Trans-epithelial Migration Assay). We include the schematic and timelines (Fig. 3a; Fig. 5a).

"Rationale for neutrophil number and 1-hour timeframe?"

Response: We now state that the dose and 1-h window were based on prior group work (Herbert et al ERJ, Robinson et al Thorax) showing robust short-interval migration without excessive apoptosis, enabling phenotyping before secondary effects dominate (Methods). We added this rationale explicitly.

“Radar plot in Fig. 1i is redundant.”

Response: We removed/reworked redundant radar plots and use radar only where it summarizes multi-marker patterns not obvious from single-marker graphs (e.g., Fig. 4f–g).

“Discussion inconsistency: prior work suggested intrinsic infant–adult neutrophil differences.”

Response: We clarified that our in-vitro work uses adult neutrophils for logistical reasons, while clinical data derive from infants. The Discussion now distinguishes peripheral blood vs cord blood contexts and notes that baseline differences (e.g., CD64) exist, but the paediatric epithelium is a dominant driver of the heightened MPO readout observed in our model (new Fig.3).

Reviewer #3

“I co-reviewed with one of the reviewers; no additional substantive points were raised.”

Response: We thank the co-reviewer for their involvement.

Response to Reviewers NCOMMS-25-82134A

We thank the reviewers for their careful assessment of our manuscript and for their constructive feedback. We are pleased that the reviewers find the system elegant and the results exciting, and we have revised the manuscript further to address the remaining concerns in detail. All changes are highlighted in the tracked changes revised manuscript.

Reviewer #1 (Remarks to the Author)

We thank Reviewer #1 for the thoughtful and detailed comments, and for recognising the strengths of the platform. We address each concern below.

Reviewer comment: The conclusion that MPO is established as a biomarker of RSV disease severity is overstated. The clinical cohort is small, heterogeneous, and sampled at variable times, and the study was not designed to validate biomarkers. The ALI system uses adult neutrophils and older paediatric epithelial cells, which limits translational claims.

Response:

We fully agree with the reviewer and appreciate this important clarification. We have revised the Abstract to closely align with the reviewer's suggested wording, removing any definitive claims regarding biomarker validation and instead emphasising MPO as a potential mechanistic readout within our model system. We emphasise that our conclusions are focused on the ability of the model to capture clinically relevant inflammatory features, and on the use of MPO as a readout within this controlled system rather than as a stand-alone clinical biomarker.

We are grateful for the reviewer's suggested abstract text, which guided these revisions and improved the clarity and precision of our claims.

Reviewer comment: The use of a single paediatric airway donor is a major limitation. Biological variability is not addressed, and the distinction between technical and biological replicates is insufficiently clear. Limitations regarding future therapeutic screening and the need for paediatric neutrophils should be acknowledged.

Response:

We agree that this represents a key limitation. All figure legends have been revised to clearly distinguish biological versus technical replicates and to specify the cell sources and exact n values used. The Discussion now explicitly acknowledges the use of a single paediatric epithelial donor and states that future therapeutic screening will require validation across multiple paediatric donors and, where possible, paediatric neutrophils.

Reviewer #2 (Remarks to the Author)

We thank Reviewer #2 for their positive assessment and for recognising the additional data and revisions that strengthened the manuscript

Reviewer #4 (Remarks to the Author)

We thank Reviewer #4 and the co-reviewer for their time and contribution
